# Social network influences and the adoption of obesity-related behaviours in adults: a critical interpretative synthesis review

**DOI:** 10.1186/s12889-019-7467-9

**Published:** 2019-08-28

**Authors:** Nestor Serrano Fuentes, Anne Rogers, Mari Carmen Portillo

**Affiliations:** NIHR CLAHRC Wessex. School of Health Sciences, University of Southampton, Building 67, University Road, Southampton, SO17 1BJ UK

**Keywords:** Social networks, Obesity, Health behaviours, Critical interpretative synthesis review

## Abstract

**Background:**

Obesity is a key risk factor for developing a long-term condition and a leading cause of mortality globally. The limited evidence associated with interventions that currently target obesity-related behaviours demand new approaches to tackle this problem. Given the evidence that social ties are implicated in the gaining and reduction of weight, the use of social networks in interventions is potentially a novel and useful means of tackling this health issue. There is a specific gap in the literature regarding what and how social network properties and processes together with environmental and individual factors influence the adoption of positive and negative obesity-related behaviours in adults.

**Methods:**

To address this gap in developing an integrated and holistic conceptual approach, a critical interpretative synthesis was undertaken following a line of argument synthesis as an analytical strategy.

**Results:**

Twenty-four studies were included. The data-driven themes *meso-micro network processes*, *contextual and individual factors*, and *types of ties and properties* were identified individually as components and causes of different health scenarios. Nevertheless, these drivers do not act on their own. As a consequence, *developing multi-agent coalitions* considering *cross-level influences* between the data-driven themes are two mechanisms that are created to understand more in-depth how social networks and the environment influence the adoption of obesity-related behaviours. These two new constructs point to a dynamic multilevel set of influences between multiple constructs, developing scenarios where positive and negative health results are identified.

**Conclusions:**

This critical interpretative synthesis offers a new means of exploring the application of social network properties and mechanisms in the ‘obesity’ field. The synthesizing argument created during the analysis process might be considered by health policy-makers, who might need to contemplate the wider open system of socially connected individuals and harness these forces to design new interventions where social networks and other contextual and individual factors operate together in a complex multilevel environment influencing obesity-related behaviours and practices.

**Electronic supplementary material:**

The online version of this article (10.1186/s12889-019-7467-9) contains supplementary material, which is available to authorized users.

## Background

Obesity is a leading public health challenge in developed and developing countries. It has reached epidemic proportions globally, with at least 2.8 million people dying each year as a result of being affected by overweight or obesity [1] and represents a key risk factor of developing a long-term condition (LTC) [2]. The estimated prevalence of individuals with obesity has increased from 921 million in 1980 to 2.1 billion in 2013 [3]. In 2016, 39% of adults aged 18 years and over had overweight and 13% obesity [4]. There are variations in incidence between countries with higher levels estimated in the United Kingdom (UK) and United States (USA) (38.2% of adults in the USA [5] and 24.8% in the UK [6]. The rise in incidence (three-fold over thirty years) has led to an increase in health services expenditure. For example, in the National Health System (NHS) the costs of treating overweight and obesity have increased from £479.3 million in 1998 to £4.2 billion in 2007 [7]. In relation to LTCs in the United Kingdom, 70% of total health costs account for this health issue [8].

A number of interventions have been designed to address obesity and overweight prevalence with the most common of these targeting obesity-related behaviours (ORBs), specifically, unhealthy food and drink choices (including alcohol), eating larger than average portions of food and a lack of physical activity [9–11]. However, a substantial number of individuals fail to adhere sustainably to these weight loss actions [12] suggesting that new approaches need to be considered to assist individuals to engage in healthy behaviours and practices in support of long-term weight loss. These approaches might usefully incorporate an understanding of the complex social and contextual influences of obesity [7, 13, 14]. Possible influences include food production, food consumption, societal influences, individual psychology and activities, activity environment and the linkages between them.

Social relationships are considered to be relevant mediators operating in open systems of a tangled and complex set of events, contexts, resources, practices, and priorities [15]. The networks of people assist in the identification of the nuanced ways in which the management of health-related practices can be integrated into open systems. Thus, people in contemporary society are mutually dependent upon one another, and relationships and connections in personal communities have considerable potential positive and negative influences on individual’s behaviours. For example, snacking habits might be shared by socially connected individuals across friends, spouses and sibling peers supporting evidence of collective behavioural process impacting on eating practices [16]. Social relationships can also influence health positively, bringing into play ‘protective’ effects. For example, in terms of physical activity, adolescent girls who have more physically active friends report higher activity levels themselves [17]. Koetsenruijter et al. [18] indicate that larger support relationships show a positive association with self-management skills in patients with diabetes, and therefore logically this applies to people with ORBs. An in-depth understanding of these relations might be useful from which to consider the design of interventions and approaches to improving obesity-related behaviours, health outcomes and associated reducing in the costs of health service utilisation. Thus, to study these potentially meaningful relationships, a network approach [19] that provides the epistemological, ontological and methodological perspective from which to understand social networks (a set of people linked to one another by specific relationships) can be applied [20]. Four dimensions of social networks are of relevance here -structure, function, strength and content [21]. The first of these considers the structural aspect of networks, including the patterns of linkages between actors [21]. Network function determines the type of exchanges, services, or supports accessible through relationships [21] whilst network strength describes the intensity and durability of ties between individuals within the network [22]. Network content refers to attitudes, emotions and behaviours flowing between network members [23]. Thus, network effects (e.g. health outcomes) are a function of interactions between these four dimensions [18].

There has been conceptual and empirical attention paid to the impact of social networks on health [24, 25]. Specifically, in terms of social networks and obesity, most of the research has focused on exploring spread, − differentiating relevant processes such as social selection, social influence and confounding effects (using mainly quantitative approaches [26]. Other authors have studied how social networks and social norms for unhealthy eating and inactivity might be associated with obesity treatments outcomes in adults [27]. Nevertheless, few network-mediated interventions have been developed to address obesity specifically. Currently, the most relevant are targeting influential individuals to spread healthy information and behaviours through interpersonal ties [28, 29] and creating opportunities for health-supportive relationships to be maintained [29–31]. In building this nascent network focus in the field, it is necessary to unpack more of the mechanisms by which social networks influence obesity with a view to developing network-based obesity interventions that alter, nurture or harness these mechanisms [32]. In order to understand how social networks affect ORBs, environmental-difference effects need to be considered [33] and understood as operating at multiple levels [34, 35]. A multilevel network perspective identifies principles that enable a more integrated understanding of phenomena that unfold within and across levels [33, 36]. Therefore, the identification, classification and integration of all these factors at different levels might develop pathways in which they are dynamically related in order to influence the adoption of positive and negative ORBs in adults and have the basis to create public health-policy relevant interventions.

The specific gap regarding what and how network properties and processes together with other factors produce positive and adverse health outcomes in adults with ORBs has been addressed to a limited extent previously [37–39]. However, there is a specific need to focus on both positive and negative results [40, 41] and for multilevel approaches to be bridged to create an integrated theory, specifying relationships between phenomena. Thus, this review aims to understand what and how social network properties and processes together with environmental-difference effects influence the adoption of healthy and unhealthy ORBs in adults.

## Methods

The complexity that surrounds the understanding of how social networks influence the adoption of ORBs requires the synthesis and interpretation of many types of different research evidence. Thus, it was decided to use a critical interpretative synthesis (CIS) as a review method, since it involves induction and interpretation of qualitative, quantitative and mixed-method data, and is primarily conceptual in process and outcome [42]. Mathematical and quantitative research powerfully describes the structure of networks and documents whether their effects are significant or not, in a statistical and theoretical sense [21]. The qualitative research presents the processes of network process and functioning, that is to say, how these networks are created [21] and what resources are transferred between them within a specific context [20].

The induction and interpretation of these data is contrary to the conventional systematic reviews which are developed as a specific methodology for assembling, pooling and summarising data [42]. Thus, this CIS aimed to generate concepts and theory where those concepts could be integrated and interpreted rather than summarising data per se. Another advantage for our research interests is that a CIS also has flexibility and is convenient in terms of appraising quality, using relevance (e.g. likely contribution to theory development) rather than methodological characteristics as a means of determining the ‘quality’ of individual papers [43]. In comparison with other methods of interpretative synthesis (e.g. meta-ethnography or grounded theory), a CIS does not only use qualitative research and, also, is distinct in its ‘explicit orientation’ towards theory generation [44], following an analysis process of different phases for the interpretation and integration of the data and, therefore, providing a more insightful way of understanding a phenomenon. The generation of a more detailed and higher-order structured theoretical framework might be useful to identify potential healthy and unhealthy scenarios.

### Search strategy

The search strategy was built around several bibliographic databases: CINAHL, Cochrane, EMBASE, Ovid, PsycINFO, Pubmed, Sociological Abstracts and Web of Science. To avoid the risk of missing relevant information, other strategies have been used to fit better with the exploratory nature of the aim [42]. This includes hand searches (between 2000 and 2017) of some key journals (e.g. *Obesity*, *Obesity Reviews*, *The Annual Review of Public Health*, *The Annual Review of Sociology* and *Behavioural Medicine*). The publication of relevant articles regarding social networks and obesity and social networks and health in these journals motivated the undertaking of specific hand searching [25–27, 32, 37, 45]. The time interval chosen was the year 2000 as there are relevant seminal papers from this date. The authors applied a citation snowballing technique to generate lists of related articles regarding the aim. Finally, specific websites were searched to identify epidemiological information related to obesity and overweight worldwide with a specific focus to the UK context, causes, prevention, obesity-related problems, relationship to prevention and management of long-term conditions, its economic impact on health systems and research institutions that are interested in social networks and obesity. These websites are *Public Health England*, *The Global Obesity Prevention Center in Johns Hopkins Bloomberg School of Public Health*, *The National Institute for Health and Care Excellence*, *Yale Institute for Network Science* and *World Health Organization*.

### Key terms

Four main terms were developed to cover the key elements of the aim of this review: obesity, long-term conditions, social networks and health behaviours (see Table 1). Diabetes-related terms were included in the list of terms connected with LTCs. The reason for this was that we found several articles regarding how social networks influence diabetes and that diabetes and obesity have several health behaviours in common, such as diet and physical activity. Also, other terms pertaining to obesity were considered but omitted finally in the search strategy such as ‘body fat’, ‘adiposity’, ‘body weight’, ‘energy intake’, ‘caloric intake’ or ‘nutrition’. This was because the focus was more on a sociological approach rather than a biomedical one. A combination of ‘all field’ search terms of each facet was undertaken to avoid missing relevant information. We applied truncations, acronyms and the booleans OR and AND to combine terms within each column and between columns from Table 1.

**Table 1:** Search strategy: key terms

Obesity	Long-term conditions	Social networks	Health behaviours
Obese Obesity Overweight	Chronic illness* Chronic disease* Chronic condition* T2DM DM DM2 Diabetes Diabetic Long-term condition* LTC Type 2 diabet*	Networks Network intervention* Peer* Peer support Social embeddedness Social influence* Social network* Social relationship* Social support	Diet Exercise Food choice Health behav* Health behav* change Health behav* intervention Physical activity Weight loss

The asterisk indicates the possible endings of some words.

### Screening

Limits were used to search the online databases: articles published in English and Spanish languages, the year of publication (between 2000 and 2017) and age groups (all adults and 19 plus years). The inclusion criteria set initially were: (i) empirical studies (qualitative, quantitative and mixed-methods) exploring the influence of relationships on the adoption of ORBs; (ii) review studies and grey literature (policy/organizational documents, conferences, abstracts). The exclusion criteria were: (i) papers that did not mention terms related to ‘relationships’, ‘social networks’ and ‘ties’ in the title or abstract; (ii) articles focused on LTCs in which ORBs were not mentioned. Eligibility of the papers was performed analysing titles and abstracts. Full papers were retrieved for independent assessment when the title and abstract appeared to meet all inclusion criteria, or when suitability could not be judged by title and abstract.

### Quality assessment and data extraction

The integration of relevance and rigor was essential in the selection of articles. ‘Rigor’ proposes that literature needs methodological credibility to address the main aim [46]. Dixon-Woods et al. [47] recommendations were followed to ensure quality assessment of the studies vis:

1. Are the aims and objectives of the research clearly stated?
2. Is the research design clearly specified and appropriate for the aims and objectives of the research?
3. Do the researchers provide a clear account of the process by which their findings were reproduced?
4. Do the researchers display enough data to support their interpretations and conclusions?
5. Is the method of analysis appropriate and adequately explicated?

Standardized data extraction templates were created to represent and make more visible data from the qualitative, mixed-methods and quantitative studies (see Table 2). They included information regarding paper reference and setting, Dixon-Woods et al. [42] appraisal prompts, methodological strengths, focus (aim) and main findings related to our research interests to illustrate the process followed.

**Table 2:** Extraction form

Type of evidence	Paper reference and setting	Dixon-Woods et al. [42] appraisal prompts for informing judgments about quality					Relevance/Rigor	Study design	Methodological strengths	Focus	Effects networks	Processes	Factors	Types of links	Parameters
		1	2	3	4	5									
Qualitative	Alvarado et al. [48]. Barbados (USA)	Y	Y	Y	Y	Y	Yes/yes	Descriptive study, semi-structured interviews	Well-described participants, provides quotes that reflect the results.	Women between 25 and 35 years, BMI equal or greater 25, not pregnant.	+/−	Social support, homophily, social modelling, diffusion, social comparison, social pressure, natural communication	Socio-cultural, psychosocial, technological, sociodemographic, environmental	Sport contacts, family, friends	
	Kennedy et al. [49]. Bulgaria, Greece, the Netherlands, Norway, Spain and UK	Y	Y	Y	Y	Y	Yes/Yes	Qualitative study, biographical interviews.	Well-described aims, sampling and recruitment and provides quotes that reflect the results	Individuals with T2DM in deprived or marginalized circumstances	+/−	Social support, social pressure, homophily	Socio-cultural, psychosocial, environmental	Healthcare professionals, community, family, friends	
	Forthofer et al. [50]. South Carolina (USA)	Y	Y	Y	Y	Y	Yes/Yes	Descriptive study, focus groups	Well-described sample and analysis	Population older than 18 years old.	+	Social support, social modelling, social pressure, diffusion	Environmental, socio-cultural, psychosocial	Family, community	Tie strength
	Knutsen et al. [51]. Bulgaria, Greece, Norway, Spain and UK	Y	Y	Y	Y	Y	Yes/Yes	Descriptive study, semi-structured interviews	Well-described methods, provides quotes that reflect the results	Individuals with T2DM from areas of high deprivation	+/−	Social support, social pressure, isolation	Socio-cultural, sociodemographic	Family, friends, community	
	Ali et al. [52]. United Arab Emirates	Y	Y	Y	Y	Y	Yes/Yes	Descriptive study, focus groups	Well-described data collection, theoretical saturation, provides quotes that reflect the results	Emirati national women, 18 years old and older with no previous diagnosis of diabetes + abdominal obesity	+/−	Homophily	Psychosocial, socio-cultural, clinical, environmental	Healthcare professionals, Housekeeping	
	Mama et al. [53]. USA	Y	Y	Y	Y	Y	Yes/Yes	Descriptive study, in -depth interviews	Theoretical saturation, provides quotes that reflect the results	Women middle-aged, obese and high socioeconomic status	–	Social pressure, social modelling, social support, homophily	Psychosocial, socio-cultural, environmental, sociodemographic	Neighbours, family	
	Robertson et al. [54]. Australia	Y	Y	Y	Y	Y	Yes/Yes	Descriptive study, in- depth interviews, semi-structured interviews and focus groups	Theoretical saturation, provides quotes that reflect the results	Overweight adults	+/−	Social pressure, social comparison, social support	Psychosocial, sociodemographic, socio-cultural	Friends, family	
	Sriram et al. [55]. USA	Y	Y	Y	Y	Y	Yes/Yes	Descriptive study, focus groups	Provides quotes that reflect the results	Midlife and older adults	+/−	Social support, social comparison, isolation, social pressure, social modelling, diffusion	Socio-cultural, environmental, sociodemographic, psychosocial	Family, friends, community, pets	
	Daborn et al. [56]. United Kingdom	Y	Y	Y	N	Y	Yes/Yes	Descriptive study, face to face in-depth interviews	Well-defined methods, provides quotes that reflect the results	Middle-aged low-income men	+	Social comparison, social support.	Psychosocial, socio-cultural, environmental, sociodemographic	Family	
	Bell et al. [57]. New Zealand	Y	Y	Y	N	Y	Yes/Yes	Descriptive study, semi-structured interviews	Provides quotes that reflect the results	Indigenous adults	+	Social support	Socio-cultural	Family	
Mixed-methods	Procter et al. [58]. United Kingdom	Y	Y	Y	Y	Y	Yes/Yes	Descriptive study, exploratory cluster randomised controlled trial and semi-structured interviews	Well-defined methods, provides quotes that reflect the results	Adults employees	+/−	Social support, diffusion, natural communication, social pressure	Sociodemographic, psychosocial, socio-cultural	Healthcare professionals, community	
	Vassilev et al. [59]. Greece, Spain, Bulgaria, Norway, United Kingdom, and Netherlands	Y	Y	Y	N	Y	Yes/Yes	Cross-sectional observational study and interviews	Well-defined methods and analysis	Adult patients with T2DM	+/−		Sociodemographic, psychosocial	Healthcare professionals	
	Koetsenruijter et al. [18]. Bulgaria, Greece, Netherlands, Norway, Spain and UK.	Y	Y	Y	Y	Y	Yes/Yes	Observational study (interviews and questionnaires)	Well described methods and analysis	Patients with T2DM	+	Social support	Sociodemographic	Family, community organizations	
Quantitative	O’Malley et al. [60]. USA	Y	Y	Y	Y	Y	Yes/Yes	Descriptive study, network survey	Well-defined methods	Adults	+			Friends	Tie strength, degree
	Conklin et al. [61]. United Kingdom	Y	Y	Y	Y	Y	Yes/Yes	Descriptive study, questionnaires	Well described sample and analysis	Adults from the European Prospective Investigation of Cancer	+/−	Social support, isolation,	Sociodemographic	Family	Tie strength
	Mötteli et al. [62]. Switzerland	Y	Y	Y	Y	Y	Yes/Yes	Descriptive study, cross-sectional study (egocentric network approach)	Well described aims and well-defined methods	Female adults	+/−	Homophily, social pressure.	Socio-cultural	Family	Tie strength
	Barclay et al. [63]. Sweden	Y	Y	Y	Y	Y	Yes/Yes	Descriptive study, logistic regressions using egocentric network data	Well-defined methods and analysis, well described aims	Adults	+	Homophily		Friends	Tie strength
	Winston et al. [64]. USA	Y	Y	Y	Y	Y	Yes/Yes	Descriptive study, cross-sectional examination of egocentric network data	Well described analysis	Black Hispanic adults with BMI more or equal 25	+	Social support		Family, co-workers	Size of the network, tie strength
	Becofsky et al. [65]. USA	Y	Y	Y	Y	Y	Yes/Yes	Descriptive study, questionnaires	Well-described methods	Adult patients	+	Social support		Family, friends	Size of network
	Shakya et al. [45]. USA	Y	Y	Y	Y	Y	Yes/Yes	Descriptive study, longitudinal survey	Well described statistical analysis	American adults	+/−	Social comparison	Socio-cultural, psychosocial		
	Rancourt et al. [66]. USA	Y	Y	Y	Y	Y	Yes/Yes	Descriptive study, ecological momentary assessment	Well-defined methods	Overweight young adult women	+	Social comparison		Friends	
	Leahey et al. [27]. USA	Y	Y	Y	Y	Y	Yes/Yes	Randomized trial	Well-defined methods and procedures	Adults with overweight or obesity	+/−	Homophily,	Socio-cultural	Family	Tie strength, size of network
	Hempler et al. [67]. Denmark	Y	Y	Y	Y	Y	Yes/Yes	Descriptive study, cross-sectional surveys	Well-described analysis	Individuals with T1DM and T2DM			Clinical		
	Christakis and Fowler [68]. USA	Y	Y	Y	Y	Y	Yes/Yes	Descriptive study, longitudinal social network analysis	Well-described analysis	Adults	+/−	Homophily	Sociodemographic	Friends	Degree of separation, social distance

### Synthesis, analysis and data extraction

The lack of an existing holistic theoretical framework of how social networks influence the adoption of ORBs motivated the selection of lines-of argument synthesis (LOA) as the analysis strategy. It implicates building a general interpretation grounded in the findings of separate studies identified by constant comparison between individual accounts and grouped in themes that are most powerful in representing the entire dataset [42]. This consisted of coding inductively empirical data, specifically, sentences from the participants’ quotes in the qualitative and mixed-methods papers and the text pertaining to the results, discussion and conclusions sections in the mixed-methods and quantitative studies (see Additional file 1). A key aspect of the analysis consisted on the synthesis and integration of the qualitative, mixed-method and quantitative data. According to the type of integration, this review is a data-based convergent synthesis design [69]. This means that all the included studies are analysed using a specific method, in this case, a qualitative synthesis method (CIS review). Since only one synthesis method is used for all the empirical evidence, data transformation is involved (quantitative data are transformed into qualitative data using categories/themes). Thus, quantitative, mixed-methods and qualitative results are presented together to answer the same objectives [69, 70]. These codes from the text and quotes from the qualitative, mixed-methods and quantitative articles (*n* = 277) represent the first-order constructs. The next step consisted of grouping these codes in wider categories, turning these into descriptive themes [71]. These data-driven themes or second-order constructs represent the original researcher’s interpretations based on first-order constructs in order to describe the content of the empirical studies [72]. In this review, three data-driven themes were created.

As it is shown in the ‘results’ section, the varied combinations and integration between these drivers can explain and modify the different results in health. The information was integrated from the studies into a coherent theoretical framework comprising a group of constructs and the relationships between them [42] producing a synthesizing argument of how social networks and the context influence the adoption of ORBs in adults. Consequently, theory-driven or third-order constructs were created built on the explanations and interpretations of the studies [42] to determine new implications for the understanding more in-depth how social networks influence the adoption of obesity and the development of future social networks interventions applied to ORBs in adults. The authors involved in the review discussed and confirmed the themes between them and other members of their research team as part of a process of reflexive dialogue against framing the analysis according to a single perspective.

## Results

**Fig. 1:**
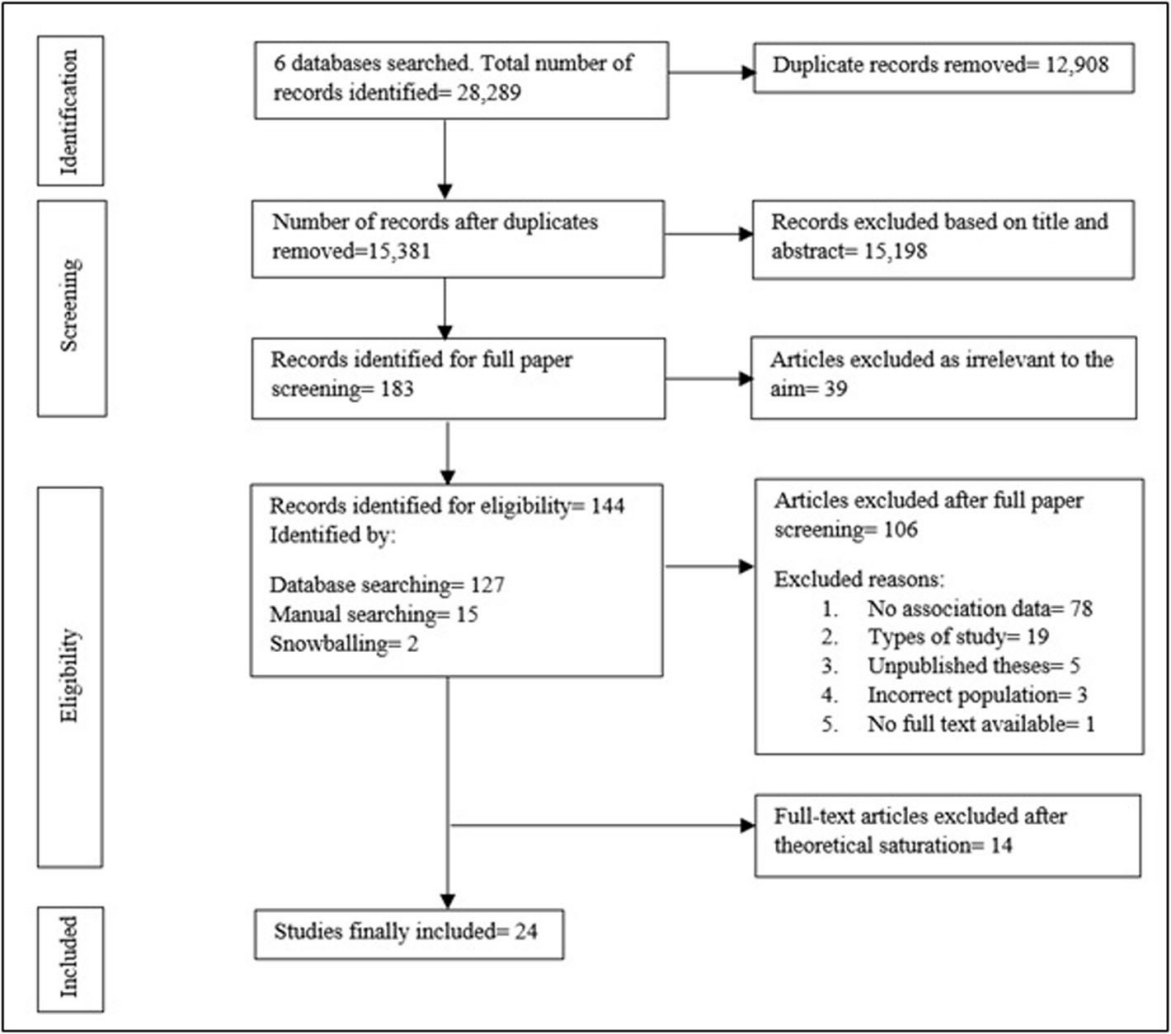
PRISMA flow of studies included in the review

After all data searches were completed 28,289 citations were retrieved, of which 12,908 were duplicates. A further 15,198 records were excluded based on title and abstract. After applying the inclusion/exclusion criteria, 183 papers were identified for full screening. After 39 articles were excluded as irrelevant to the aim of the CIS, 144 papers were identified for eligibility (127 from database searching, 15 from manual searching and 2 from snowballing). Of the 144 studies screened, 106 were excluded after a full paper screening and 14 after theoretical saturation [73]. Through the theoretical sampling, qualitative (*n* = 10), mixed-methods (*n* = 3) and quantitative studies (*n* = 11) were included and reviewed until theoretical saturation was achieved [47, 73–77] (see Fig. 1). Theoretical saturation is the point at which additional data does not lead to any emergent themes or concepts in the analysis [78–80]. It was relevant here as broad spectrum criteria for the inclusion and exclusion of identified relevant articles. In this review, theoretical saturation signalled the end of the identification of articles in which social relationships were related to the adoption of ORBs in adults. It needs to be highlighted that although grey literature was also reviewed, it was not included in the final analysis of review findings since it did not provide sufficient relevant information on the topic. Thus, 24 articles were theoretically rich enough and of central relevance to the aim.

### Data-driven themes

Three data-driven themes were developed from the reviewed literature: *meso-micro network processes for the adoption of ORBs, contextual and individual factors of ORBs, and types of ties and properties that influence the adoption of ORBs.*

The abstraction of the inductive concepts covered two levels of analysis. The following levels of analysis with their initial characteristics are adapted for this article based on relevant research of other authors [35, 75, 81–85]:

- *Meso-level:* describing relationships with healthcare professionals and other individuals from the community or locality (neighbourhood, suburb, city).
- *Micro-level:* examining a person’s closest social circle-peers (family, friends, co-workers, pets) and individual characteristics (biological, psychological and personal history factors).

#### Meso-micro network processes for the adoption of obesity-related behaviours

Network processes are responsible for tie formation and outcomes [86] located within a social context that shapes practices, behaviours and the roles and functions that networked individuals achieve [49]. These network processes are classified into the different levels of analysis according to the nature of and involvement of ties participating in the relationships.

The reviewed papers identified network processes at the *meso and micro* levels of analysis. Regarding the *meso-level*, the most relevant processes were *social support* [48, 49, 51, 55, 58] (Table 3, Q1 and Q2) and *social pressure* [48, 50, 51, 55, 58] found in 5 articles which have both positive (Table 3, Q3) and negative effects on health. For example, in terms of positive effects, a conversation in the streets with a friend from the gym might exert positive social pressure and increase the motivation to exercise [48]. With regard to negative effects, individuals with specific cultural norms might for instance influence the quality and amount of food intake (Table 3, Q4). Another relevant network process is *homophily* [48, 49, 52, 68], the tendency of pairs of individuals to share the same characteristics [20], described as a positive network process to exercise -e.g. when individuals share the same gender (T1 in Table 3). The *diffusion* of new health information is also linked to positive effects on the adoption of ORBs (see Q6 in Table 3).

**Table 3:** Data-Driven Themes abstracted from quotes and text of the articles

Data-Driven Themes	Quotes and Text From Findings
Meso-micro network processes	Q1(+). “Several participants suggested external health promoters could provide additional encouragement: ‘Somebody coming in from outside, say doing half an hour at lunchtime just doing a presentation about it or, you know, longer and getting people there and talking about that and saying ‘and we have our in-house person who you know if you want to talk to him, d’you wanna get encouragement from him/her’ that would be great but I think somebody coming in from outside actually would be a good idea.” [58] Q2(+). “That’s why I think the group would be kind of cool to get together with… to get together as a group and just share some ideas ...” [55] T1(+). “During follow-up visits all but one woman in the study agreed that men and women were not active together. In contrast, women reported discussing exercise with other women and joining all-female exercise groups.” [48] Q3(+). “I probably would pass somebody from my gym somewhere on the streets [… It’s] motivational in the sense that if you don’t go […to the gym] and pass a girl that I haven’t seen in a while ‘hey why I don’t see you in the gym? What’s going on with you?’ and I guess guilt people into coming back. So yeah it’s motivation.” [48] Q4(−). “However, if I go back to the village where my husband comes from, they are country people and they love to bake, cook and it’s lovely. It’s gorgeous and because they know you’re coming for afternoon tea, they’ll have made you the apple pie and they’ll have made you the cakes and if you went in there and tried to start explaining that you don’t eat any of that… In that sort of culture, it just would not be understood, and also you’re interfering with the social norms and you don’t want to do that.” [51] T2(+). “WhatsApp groups comprised of women in the same exercise class could make this social pressure and social support even stronger.” [48] Q5(+). “We have people in my neighbourhood that you can be leaving out at five in the morning, and they’re walking. You can come in at six in the afternoon and there’s another group walking…We have a monthly HOA [homeowners association] meeting— and sometimes in those meetings people just go, “Hey, I saw you walking. Can I join your group?” [53] Q6(+). “Overall the promoters found their booklet ‘was well set out’ and helped them approach participants: ‘It was informative and useful and helped me set out what I needed to do, promote walking to work to the colleagues, and how to approach them and stuff, I thought it was quite good.” [58] T3(+). “In contrast, having tight social networks was viewed as beneficial if friends were “health-conscious” and acted as positive role models.” [55] T4(+). “In the case of body size, a descriptive norms effect can work through direct comparison so that a person compares himself to others in his social reference group and makes decisions regarding his own status according to that metric.” [45] Q7(+). “My husband insists that I shouldn’t eat large quantities or any starchy food. My mum always scolds me, but this doesn’t help; she just gets on my nerves. As soon as she sees me eating even the smallest amount of sweets, she’ll start complaining. I can’t say my daughters are indifferent. They’ll remark when I overeat something. Everyone is focused on my diet.” [51] T5(−). “Social events involving food were areas where maintaining normal social ties were often more important than attempting to force attention on dietary needs.” [49] Q8(+). “I used to eat a lot of vegetables when I was at home, cause my wife was an extremely good cook, so we ate really well, I don’t mean gluttony I mean just healthy food.” [56] Q9(+). “We are trying to exercise together, all of us… We aim to create a large group and include family and kids and socialize very often, so it becomes a big group and better habits.” [49]
	T6(+). “More precisely, women and their most important eating companions tended to be similar in diet-related factors such as diet quality and eating styles as well as in BMI.” [62] Q10(+). “…found myself you know doing the walking home without having written it down and you know having told several people – I mean telling people that that’s what you’re doing actually makes you hold to it even more than if you, if I’d written it down.” [58] T7(−). “Several elderly women also discussed the negative consequences of living alone on their diets. Without family members around, eating decisions were primarily based on convenience and several participants reported having no incentive to make dietary improvements at their advanced age.” [55]
Contextual and individual factors	Q11(−). “Close to my house, there are no sidewalks. And I feel like I don’t want to get in the car and drive somewhere and get out and walk and get back in the car… I used to walk a lot, but I lived somewhere else so it made it very simple.” [50] Q12(+). “I’m looking for more, all the time… I’m getting ideas at the moment because when I go to the sports centre they’ve got loads of activities for older people like me and other illnesses, not just diabetes, they cover everything there.” [49] Q13(−). “When the weather is cold I walk, but it is difficult to walk in summer.” [52] T8(+). “Participants described food-centric social events as a primary constraint to eating well. Limited entertainment options in these rural communities meant that most activities involved getting together for a snack or meal. Food provision was regarded as a sign of “hospitality” and people felt obligated to eat whatever was offered in social settings (e.g., church, senior centers).” [55] Q14(−). “Walking is not culturally acceptable. My husband will not allow me to walk in the street but if it is a closed place [gym] he has no problem.” [52] Q15(+). “Going to the gym the motivation is, well obviously it would be generally to lose weight, but going by the gym is relatively small so you know everybody that is there so it’s kind of a family type atmosphere.” [48] Q16(+). “My grandmother… when I was 13… I was the sole witness to her coronary occlusion which killed her on the spot and I never quite dealt with that so it has left me with a bit of a fear of heart disease and heart problems and seeing how violently they can end your life.” [56] Q17(+). “There is some type of apprehension in the back of my mind, and I’m trying to figure out why, but I really need to say, “Go ahead, start doing it.” I guess I feel that if I start, I’m going to have to continue. It’s going to change my routine. [And that] Moves me out of my comfort zone.” [53] Q18(−). “I have the control to change things I just don’t change them, and I don’t know why. It’s ridiculous.” [54] Q19(−). “The food we eat is not healthy because of the way we cook it and because we do not know enough about healthy food.” [52] Q20(−). “I think people [study participants] have the intentions of walking … but, because their character is, just they don’t know how to live without the car.” [58] T9(−). “During treatment, participants lost an average of 4.4% of initial body weight, and social influence factors were adversely associated with weight loss outcomes. Having more casual friends who were overweight at baseline and being part of a social network with stronger social norms for unhealthy eating predicted poorer weight losses (p’s < .023).” [27] Q21(−). “I look after my husband, the house, everything. I don’t look after myself as much as I used to. In the past, I would cook something for myself and something for the others to eat… I have to cook meals that my children and grandchildren like because my daughter works, and so I eat from these as well, so I don’t miss out.” [51]
	T10(−). “This man emphasizes the need to occasionally not adhere to the diet, especially at parties and when with friends.” [51] Q22(−). “Without the help of my children, I wouldn’t be able to cope. My pension is 140 leva—[not enough] for following a diet and buying drugs.” [51] Q23(−). “I worked as […] a cashier at a supermarket until 2009 and you know a cashier sits down ain’t much activity in that and then in 2009 to 2011 I did secretarial work – so that’s even worse but then […] I got this new job that I totally love cause since I really can’t get the exercise that I want to put in…” [48] T11(+). “Specifically, people who reported good self-management skills were more likely to have a diverse network, to be older, to be in relatively good health, to have high levels of income and education, and to live in the wealthier of the six countries (Norway, UK, Netherlands, Spain). High levels of self-monitoring were also associated with high education and relatively good health.” [59] T12(+). “In these small rural towns, social interaction appeared to be an important facilitator of active lifestyles, particularly for women. Organized group activities, such as walking, were viewed as an opportunity to socialize with friends and connect with the community. Building these networks increased enjoyment and gave people more incentive to engage in activity.” [55] Q24(−). “Sometimes the walk is be good you know exercise but if I have my car I wouldn’t walk at all only when I don’t have do I walk cause everything closer in town [Bridgetown] that ya could walk to instead of wasting the gas but as for out here [St. Philip]… the closest shop there … nah… now that is daytime no way! Ain’t walking. Too hot!” [48] T11(+). “Specifically, people who reported good self-management skills were more likely to have a diverse network, to be older, to be in relatively good health, to have high levels of income and education, and to live in the wealthier of the six countries (Norway, UK, Netherlands, Spain). High levels of self-monitoring were also associated with high education and relatively good health.” [67] T13(−). “People with type 2 diabetes were less physically active, less likely to follow recommended diet (men), had fewer contacts with family and friends and were less certain of counting on help in case of severe illness than people with type 1 diabetes.” [67]
Types of ties	Q3(+). “I probably would pass somebody from my gym somewhere on the streets [… It’s] motivational in the sense that if you don’t go […to the gym] and pass a girl that I haven’t seen in a while ‘hey why I don’t see you in the gym? What’s going on with you?’ and I guess guilt people into coming back. So yeah it’s motivation.” [48] T14(+). “In Bulgaria compared to elsewhere, health professionals’ advice was taken more seriously and sought more frequently.” [49] Q4(+). “We have people in my neighbourhood that you can be leaving out at five in the morning, and they’re walking. You can come in at six in the afternoon and there’s another group walking… We have a monthly HOA [homeowners association] meeting— and sometimes in those meetings people just go, "Hey, I saw you walking. Can I join your group?” [53] T15(+). “Attending community organizations was positively related to physical activity, however only for patients with a low income (OR = 1.53).” [18] Q25(+). “…my health…[is] my family…My children and husband, and our whanau whanui (tribal family) … our wellbeing is whanau (family)…[when] someone else is not well in our family, that has an impact…on our health…I’m connected to those people and our children…the heavier we are collectively, the better off we are individually…” [57]
	Q26(−). “No, my family doesn’t help me. I am responsible for health issues at home… I ask them to support me a bit more, taking the cakes out of my sight, but they’re all tomboy-like and take little care of me. They don’t see a disease in my diabetes.” [49] T16(+). “Whether the target was a friend moderated these effects. When engaging in an upward comparison to a friend, participants had more thoughts of exercising compared to when the target of the upward comparison was not a friend (Y = 1.03, *P* = 0.031). When engaging in a downward comparison to a friend, participants also reported more thoughts of dieting (Y = 2.68, *P* = 0.006) and exercising (Y = 2.13, *P* = 0.024) as compared to when targets were nonfriends.” [66] T9(−). “During treatment, participants lost an average of 4.4% of initial body weight, and social influence factors were adversely associated with weight loss outcomes. Having more casual friends who were overweight at baseline and being part of a social network with stronger social norms for unhealthy eating predicted poorer weight losses (p’s < .023).” [27] Q27(−). “Our weights increase because we have housemaids and we depend on them a lot.” [52] T17(+). “In a multivariable regression model, greater weight loss was associated with help from a child with eating goals (*p* = .0002) and co-worker help with physical activity (*p* = .01).” [64] T18(+). “For several participants, pets provided much needed companionship and reason to be active. Pets appeared to be especially important motivators of physical activity for elderly individuals living alone.” [55]
Properties of social networks	T19. “Lower frequencies of family contact were associated with lower fruit variety scores and rare/no contact was similarly negative for both genders. By contrast, decreasing family contact seemed to have limited association with vegetable variety in men whereas weekly contact had a 0.56 unit difference (p ¼ 0.001) in score in women compared with daily family contact.” [61] T20. “The degree to which this behaviour is shared is modulated by the strength of the relationship between the two individuals, with a greater probability of engaging in these behaviours observed when the relationship with the nominated peer is strong relative to when the relationship is weak.” [63] T21. “Moreover, having more friends is associated with an improvement in health, while being healthy and prosocial is associated with closer relationships. Specifically, a unit increase in health is associated with an expected 0.45 percentage-point increase in average closeness, while adding a prosocial activity is associated with a 0.46 percentage-point increase in the closeness of one’s relationships.” [60] T22. “Participants reporting social contact with 6 or 7 friends on a weekly basis had a 24% lower mortality risk than those in contact with ≤1 friend (HR 0.76, 95% CI 0.58–0.98).” [65] T9. “During treatment, participants lost an average of 4.4% of initial body weight, and social influence factors were adversely associated with weight loss outcomes. Having more casual friends who were overweight at baseline and being part of a social network with stronger social norms for unhealthy eating predicted poorer weight losses (p’s < .023).” [27] T23. “Whereas increasing social distance appeared to decrease the effect of an alter on an ego, increasing geographic distance did not. The obesity of the most geographically distant alters correlated as strongly with an ego’s obesity as did the obesity of the geographically closest alters. These results suggest that social distance plays a stronger role than geographic distance in the spread of behaviours or norms associated with obesity.” [68]

The *micro-level* is represented more than the *meso-level*-mentioned in 21 articles [18, 27, 45, 48–58, 61, 62, 64–66, 68] with 13 articles identifying *social support* as an essential network process for the adoption of healthy practices [18, 48–51, 53–57, 64, 65]. As *social support* is a broad concept, it was sub-divided. *Peer support* by family members was key to acquiring good dietary habits [56] (Table 3, Q8) and *group support* to performing physical activity [49], as of Q9 in Table 3. *Homophily* [62] (Table 3, T6) and *social comparison* [45] with families and friends also representing positive processes for the adoption of healthy ORBs in adults. For instance, as shown in T4 in Table 3, individuals might make decisions about their own health whilst they are comparing their body size with others. The presence of negative effects on health included *isolation*, described as a high-risk factor of developing unhealthy behaviours [51, 55, 61] and a variety of diet (Table 3, T7). The network facet of *social pressure* showed dual effects [49, 51, 53, 54, 62]. For example, a negative effect is when friends push the individual not to follow the diet in the context of a social event (Table 3, Q7 and T5). A positive effect is the pressure that the family exerts on the individual’s diet (Table 3, Q7) or when a friend encourages the individual to exercise [53]. Finally, *natural communication* [48, 58] and *social modelling* [50, 53, 55] were described in the reviewed literature in both levels of analysis as processes for the adoption of positive ORBs, as illustrated in T2, Q5, T3 and Q10 in Table 3.

#### Contextual and individual factors for the adoption of obesity-related behaviours

In addition to *meso-micro network processes*, there is evidence that shows how other *contextual and individual factors* influence the adoption of ORBs in adults.

With regard to the *meso-level*, *environmental factors* described in 7 articles [48–50, 52, 53, 55, 56] the lack of conducive built environments were considered to be the main barriers to exercise (Table 3, Q11), whilst community resources such as group activities stimulate the adoption of healthy activities in older people (Table 3, Q12). Additionally, *socio-cultural factors* [48, 49, 51, 52, 55, 57] were considered a positive and negative influence on normative social responses to individuals’ behaviours. For example, regarding negative effects, gendered norms are included in some communities which may impact through presenting a barrier to be overcome in order to perform physical activity (Table 3, Q14).

Eighteen articles described *contextual and individual factors* at the *micro-level* [18, 27, 45, 48–56, 59, 62, 63, 67, 68, 87] and 11 articles showed that *psychosocial factors* [45, 48–50, 52–56, 58, 59] play an essential role in the adoption of ORBs. For example, regarding positive effects, living through *critical moments* like the loss of a relative [56], possessing high internal *motivation* [53], *self-efficacy* [54] and a specific *knowledge* about healthy food [52] are facilitators to change health behaviours, as stated in Q16, Q17, Q18 and Q19 in Table 3. Nevertheless, *personal attitude* is sometimes a barrier; this is the case when a person decides between the use of the car or walking to commute (Table 3, Q20). Moreover, *socio-cultural factors* were described in 13 articles [27, 45, 48–56, 58, 62], showing negative effects on health in most of the examples. This is the case concerning *social norms* [27], *social events* [51] and *competing demands* [55] such as family responsibilities (caring, children hobbies). *Sociodemographic factors* were identified in 11 articles [18, 48, 51, 53–56, 58, 59, 61, 68] with *socio-economic status* [18, 48, 51, 54, 56, 59] and *gender* [54–56, 59, 61, 68] having positive and negative effects. A poor socio-economic status with a lack of social support might be a barrier to accessing healthier food in following a healthy diet (Table 3, Q22). On the other hand, having high levels of income is associated with better education and the self-report of good self-management skills (Table 3, T11). In terms of *gender*, women are seemingly more willing to participate in community groups and work with a shared aim collectively than men (Table 3, T12). Certain types of *jobs*, *age*, *transport* and *education* were also mentioned (Table 3, Q23, T11 and Q24). *Clinical factors* such as underlying medical conditions are barriers to performing physical activity [67]. For example, individuals with type 2 diabetes are more willing to exercise than individuals with type 1 diabetes (Table 3, T13). This may be because overweight and obesity, which in many cases, accompanies type 2 diabetes, might be the precipitating factor.

#### Types of ties and properties of social networks for the adoption of obesity-related behaviours

In this review, *ties* are understood as the links between individuals [20]. They are meaningful as different types of contacts offering potential ways to change behaviours.

Ten articles described *weak ties* at the *meso-level*. The strength of ‘weak ties’ hypothesizes that things flow from people with whom one has limited tenuous contact and relationality [87]. Here, *community organizations and community groups* [18, 49–51, 55, 58] were identified as relevant ties for the adoption of a good diet and physical behaviours. Also, networking with *healthcare professionals* and *neighbours* were identified as a means of getting involved with behaviour change, as illustrated in T14 and Q4 in Table 3. Twenty articles described contacts at the *micro-level* as key players for the adoption of ORBs. These included in the main strong ties, that is to say, relations between contacts that have strong bonding connections between them. In this regard, 15 articles related *family* contacts with positive and negative effects on health [18, 48–57, 61, 62, 64, 65]. For example, there may be cases where family members might be supportive or not with diet (Table 3, Q25 and Q26). *Friends* were also mentioned in 11 articles as key players [27, 48, 49, 51, 54, 55, 60, 63, 65, 66, 68]. It is seemingly easier to engage in exercising if individuals compare themselves with friends [66] (Table 3, T16). On the other hand, there is a high risk of adopting bad dietary habits if a person belongs to a group of friends with overweight and with a strong set of social norms for unhealthy eating, as stated in T9 in Table 3. Finally, according to the literature *sports contacts* [48], *housekeeping* [52], *co-workers* [64] or *pets* [55] are identified as types of ties at the *meso-micro levels* of analysis (Table 3, Q3, Q27, T17 and T18).

Networks are formed by individuals and the ties among them and thus, it is relevant to continue the description of networks by examining simple properties or measurements of these social structures [86]. Some key features of the social networks were essential to define the structure of the network and patterns of the adoption of ORBs in adults. More concretely, in 7 articles [27, 50, 60–64] the *tie strength (frequency of contact and feeling of closeness)* with members of the family or healthy companions consistently describes higher diet quality as it can be seen in T19 and T20 in Table 3. Another property to consider is the *degree* or *number of contacts* that each individual possesses. Having more friends is related to healthier habits (Table 3, T21). Moreover, the *size of the network* is relevant for positive and negative effects [27, 64, 65]. The bigger the network an individual is part of with poor health habits, the harder it might be to lose weight (Table 3, T22). By contrast, individuals reporting social contact with six or seven friends have more opportunities to engage in healthy behaviours than those in contact with only one friend (T9 in Table 3). Finally, distances between members of the network were considered in terms of *social distance (degree of separation)* and *geographic distance.* The *social distance* is the distance that an individual is from others [88] (e.g. the distance between two adjacent individuals is one). A study of the spread of obesity in a large social network over 32 years demonstrated that increasing *social distances* appeared to decrease the influence of contacts to enhance weight gain in the individual, but increasing *geographic distance* did not [68], that is to say, the *geographical distance* did not modify the effect of those contacts. Thus, *social distance* could play a stronger role than *geographic distance* in the spread of behaviours or norms associated with obesity.

### Theory-driven themes

**Fig. 2:**
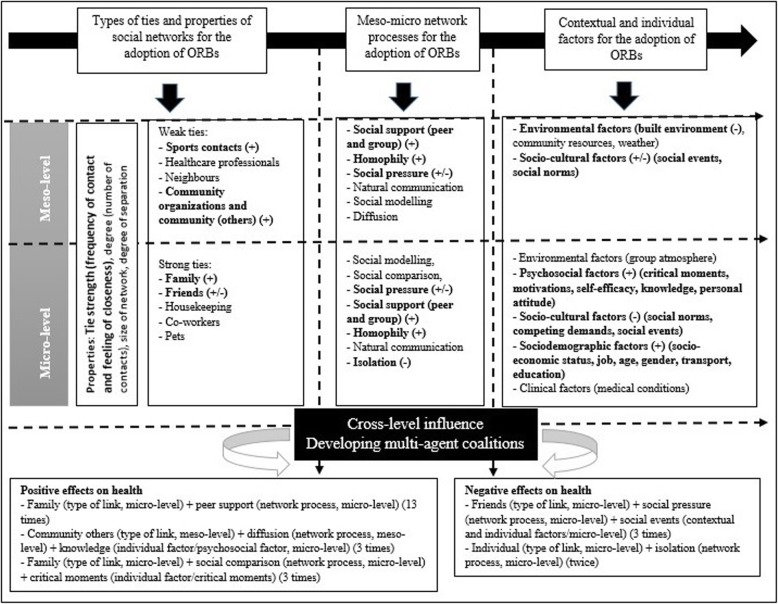
Complex system approach with inductive constructs. (Legend) The most relevant constructs are highlighted in bold. (+) and (−) symbols explain if the constructs have positive or negative effects on health. The two theory-driven themes point to different coalitions with positive and negative effects on health

The aim of the review required an in-depth exploration of how data-driven themes influence the adoption of positive and negative ORB’s in adults. Two theory-driven themes have been developed from the synthesizing argument to complete this theoretical framework, and the understanding of the events studied (see Fig. 2). These themes are *developing multi-agent coalitions* and *cross-level influence*.

In the process of the review, different health scenarios where social networks are present in the adoption of ORBs have been broken down to different levels of analysis. In each scenario (text or quote), various components have been identified and coded individually, showing their positive and negative effects on health. They were then grouped in wider categories or data-driven themes. These themes were *meso*-*micro network processes*, *contextual and individual factors* and *types of ties and properties of social networks*. Nevertheless, during the process of analysis, it has been shown how the majority of the components cannot act on their own but were mediated in some way to affect health. Thus, *developing multi-agent coalitions* is a driver that highlights the necessity of the combination and influence between these different concepts, developing power of cumulative effects. These different *multi-agents coalitions* produce and, therefore, acknowledge both positive and negative health outcomes in the individuals. For example, *social pressure* by close contacts (*friends or family*) at *micro-level* has shown typically positive results in this review [51, 53, 54, 62]. Nevertheless, if a *socio-cultural factor* (e.g. *social events*) is considered, this effect might be more harmful. In this latest case, it is shown how a positive effect is reversed into a negative one by adding an extra component (*contextual factor*). The following two examples represent this fact.

*“My husband insists that I shouldn’t eat large quantities or any starchy food. My mum always scolds me, but this doesn’t help; she just gets on my nerves. As soon as she sees me eating even the smallest amount of sweets, she’ll start complaining. I can’t say my daughters are indifferent. They’ll remark when I overeat something. Everyone is focused on my diet.”* [51]

This example represents, a *multi-agent coalition* culminating in a positive effect on health*: Family (type of tie, micro-level) + social pressure (process, micro-level).*

*“This man emphasizes the need to occasionally not adhere to the diet, especially at parties and when with friends.”* [51]

This last example, which was extracted from the same article as the previous one, shows how adding an extra data-driven theme (party, *social event*) changes the effect into one, which is more negative. This coalition would be *friends (type of tie, micro-level)* + *social pressure (process, micro-level)* + social events *(contextual and individual factors/socio-cultural factors, micro-level)*.

The identified relevant combinations implicating positive and negative effects on health are available in Additional file 2. The most relevant *multi-agent coalitions* are presented below, based on the number of times that appeared in the chosen articles and classified into positive and negative health effects.

Positive effects:

- *Family (type of link, micro-level) + peer support (network process, micro-level)* (13 times).
- *Community others (type of link, meso-level) + diffusion (network process, meso-level) + knowledge (individual factor/psychosocial factor, micro-level)* (3 times).
- *Family (type of link, micro-level) + social comparison (network process, micro-level) + critical moments (individual factor/critical moments)* (3 times).

Negative effects:

- *Friends (type of link, micro-level) + social pressure (network process, micro-level) + social events (contextual and individual factors/micro-level)* (3 times)*.*
- *Individual (type of link, micro-level) + isolation (network process, micro-level)* (twice)*.*

*Cross-level influence* refers to the dynamic relationship between the individuals that are embedded in social relationships and the context at multiple levels of analysis, in this case, *micro* and *meso* levels. This concept cannot be understood without the influence of *developing multi-agent coalitions*. Two examples with the involvement of different levels of analysis are illuminating:

*“I probably would pass somebody from my gym somewhere on the streets [… It’s] motivational in the sense that if you don’t go […to the gym] and pass a girl that I haven’t seen in a while ‘hey why I don’t see you in the gym? What’s going on with you?’ and I guess guilt people into coming back. So yeah it’s motivation.”* [48]

This event is formed by a *type of link (sports contacts, meso-level)*, a *network process (social pressure, meso-level)* and an *individual factor (psychosocial factor/motivation, micro-level)*.

*“Participation and attendance at the pub involve negotiations and a counterbalance of the intake of beer and the health promoting effects of positive social relationships taking place at the pub.”* [51]

This coalition can be interpreted as the combination of *friends (type of tie, micro-level)* + *peer support or group support (process, meso-level)* + *social norms and social events (contextual factor/socio-cultural factor, meso-level)*.

Thus, as per Fig. 2, it can be seen that *developing multi-agent coalitions* and the presence of *cross-level influence* between the different data-driven themes produce positive and negative ORBs.

## Discussion

This CIS offers an opportunity to gain novel insights regarding how social networks influence the adoption (or abandonment) of positive and negative ORBs in adults with obesity, overweight or risk of obesity. The qualitative, quantitative and mixed-method empirical evidence from the reviewed papers were included in the process of the development of new themes and a synthesizing argument with the aim of addressing a gap found in the literature namely what and how network properties and processes together with other environmental factors produce positive and negative health outcomes in adults with ORBs.

The consideration of all dimensions of network analysis and exploration, that is to say, structure, function, strength and content required the contribution of both qualitative and quantitative approaches. Traditionally, theories have identified a variety of processes in social networks and obesity research, generating discussions between researchers about aspects of social selection, social contagion, confounding, social influence [32, 68, 89, 90]. Here our focus is mainly on social influences, which have been classified under the umbrella term of social processes in order to open up new ways of discussion about the underlying processes of social networks.

A strength of this article was the application of social network theory which in previous studies identified the importance of relationships in the adoption of ORBs but without considering this network approach [48, 52–57, 61, 62] in a manner which illuminates the depth, meaning and structure of these relationships and associations with contextual and environmental factors. This affirms that structural and functional characteristics of social networks together with environmental and personal factors may contribute to health behaviours [91–93]. Several authors have recognized some of these ideas before, but individually and in the absence of considering the sum of other factors in the adoption of health behaviours [37–39, 94, 95]. This review presents an integrated developed contribution in comparison with the previous studies, in particular flagging up how the combination and the relationship between these concepts at different levels of analysis produce positive, negative and contingent health behavioural outcomes in adults with ORBs. Thus, different agents were identified at two levels of analysis (*meso* and *micro* levels). This research resonates with Bronfenbrenner’s ecological theory [35] since it was concerned with tackling numerous environmental factors and numerous persons in different interaction relationships, roles, actions and processes. Nevertheless, the formula of using the six different levels of analysis (*individual*, *microsystem*, *mesosystem*, *exosystem*, *macrosystem* and *chronosystem*) do not equate with our idea about open system thinking, which is more related with the multilevel approach of social network theory. Thus, the levels of analysis are simplified into, *micro*, *meso* and *macro* levels [81, 96]. In this regard, networks are understood as a dynamic response to individual interactions [97]. It implies that social interaction is actually the most elementary unit of social belonging and dynamics, and thus that it is what generates social spaces and positions [98, 99]. Following this, we cannot consider in the same level of analysis (e.g. *micro-level*), for example, the role of family and health services, since the interaction of the individual with them might be potentially different. Similarly, in the *micro-level* we have identified the potential contacts that have a stronger and closer relationship with the individual. In the *meso-level*, community contacts and less-frequent contacts are situated. This way of thinking is in line with other authors’ research [62, 63]. Also, we have decided to propose the *meso-level* as the limit of the analysis because of the relevant amount of available.

The main focus of this CIS is to show how networks, together with the environment, influence the adoption of ORBs. During the process of analysis of data, different health scenarios were broken down into different themes at different stages. This process allows the creation of a ‘story’ (synthesizing argument) that explores ‘what’ and ‘how’ these new themes influence the adoption of ORBs. As a consequence, two new synthetic constructs *developing multi-agent coalitions* and *cross-level influence* point to a dynamic multilevel set of influences between multiple constructs (data-driven themes) that produce different positive and negative health results. With regard to positive effects, the combination of *family* (*type of link, micro-level*) with *peer support* (*network process, micro-level*) is the most important because it was the most mentioned in the literature (thirteen times), showing the engagement of individuals in health behaviours. By contrast, with regard to negative effects, the combination of *friends* (*type of link, micro-level*), with *social pressure* (*network process, micro-level*) and *social events* (*contextual and individual factors, micro-level*) is the most relevant (appearing three times). In relation to this, a relevant aspect that has been uncovered during the review is the potential power that the data-driven themes possess to address or reverse unwanted effects in which context plays a significant role. For example, *social pressure* by *family* at *micro-level* has typically positive effects on health. This is the case when the *family* control what an individual is eating at home. Nevertheless, *social pressure* exerted by close contacts in concrete *social events* (e.g. parties, pubs) enhances the adoption of negative ORBs, such as the intake of unhealthy food.

These results could have utility for health policy, considering the design of innovative interventions based on the integration of social networks and other contextual factors at multiple levels of analysis. The *development of multi-agents coalitions* between the different individual components of the data-driven themes created, using *cross-level influences* might be suitable to apply in the complex environment where individuals live. Thus, per example, this review has shown that the coalition *family* (type of link, micro-level) with *peer support* (network process, micro-level) has potential positive influences on health. In this sense, it would be interesting to explore the design of interventions where different members of the family could be embedded. For example, delivering educational programmes to the family rather than the individual or the collective participation of the family in different community assets and activities. This might enhance the influence of other members in the community and the exertion of group support (e.g. running group). In these examples, different aspects of networks together with contextual factors are present. The use of social network online tools based on social prescription and acknowledgement of new resources instead of the typical and well-known resources of the community to support this engagement with self-directed support might be relevant. Social network online tools might be used to identify other members of an individual network (apart from the family) that might be interested in sharing these activities with other people, even to connect ‘isolated’ individuals to others and these community assets or activities. This would potentially increase the size of the social network to avoid isolation and loneliness in adults, both risk factors for mortality associated with obesity. Isolation has been identified in this review as one of the main issues in ORBs. The most relevant scenario of negative health effects in this CIS was the combination of *friends (type of link, micro-level)*, with *social pressure (network process, micro-level)* and *social events (contextual and individual factors, micro-level)*. In this sense, it has been demonstrated in the results section how different constructs such as the *contextual factor ‘social event’* has the power to reverse positive health effects. From a health policy perspective, it might be relevant to modify *contextual and individual factors* such as paying greater attention to how we can create or modify infrastructures and environments to practise physical activity or enhance the self-efficacy of avoiding unhealthy practices (e.g. alcohol intake, high-sugar food) in social events. The consideration of attending specific social events (e.g. regular meetings in pubs), in which the relationships influence negatively, might be relevant for predicting potential negative results. The regulation and limitation of alcohol intake or an increase in its price in local and macro festivals and pubs (places where social relationships enhance their consumption) can be other actions. Additionally, the identification and visibility of influential individuals (e.g. friends that go to the gym regularly or celebrities that promote healthy cooking in the media) might be considered as a prominent mediator in engaging people who wish to make changes to their health behaviours and their social norms.

Consideration needs to be given to the nature of qualitative research and the understanding of the open social systems analysed in this review, suggesting typicality rather than the generalization of the data abstracted [100]. These results might not be reproducible and predictive against the different criteria used in quantitative research. The creation of the synthesizing argument with the results obtained in this review provides a novel and more conceptually deep starting point for future interventions, considering conceptualizations at multiple levels for theoretical and application-relevant interventions which quantitative studies alone are unable to provide. In order to explore the veracity content and nature of the ties and the specific contexts where these relationships occur, it is necessary to translate and use these finding in the design of interventions.

This CIS has limitations. Firstly, the findings contained in the included studies were interpreted according to our research interests. The lack of studies regarding how mechanisms and properties of social networks influence the adoption of health behaviours, in general, opens an easily questionable route of interpretation. In this sense, the transformation of quantitative data into a qualitative form followed a ‘coding’ process. The use of this analysis strategy to deal with quantitative data could be questionable due to the lack of reviews that integrate qualitative and quantitative data with which to compare. Another limitation is the restriction on searching only in the English and Spanish language. It may have excluded relevant articles. However, the requirement of translation could result in the misinterpretation of specific information. We are conscious that the final number of articles that explicitly identified the specific focus of social networks and obesity-related behaviours were limited in number. Nevertheless, these were sufficient to attain theoretical saturation [47, 73–77]. In this sense, concepts and linkages between them were well-developed, and no additional data were needed. Finally, the authors are aware of how broad and complex the topic is. Although the aim is to provide a whole and innovative vision of an event, certain factors might require more in-depth analysis using other strategies and more empirical work.

## Conclusions

This CIS offers a new way to understand the use of social networks in the ‘obesity’ field in open settings. Breaking down different health scenarios in an analytical process allows the creation of a synthesizing argument that explores ‘what’ and ‘how’ social networks together with environmental-difference effects influence the adoption of positive and negative ORBs in adults using a multilevel approach. The data-driven themes *meso-micro network processes*, *contextual and individual factors*, and *types of ties and properties* were identified individually as components and causes of different health scenarios. Nevertheless, these drivers do not act on their own. As a consequence, *developing multi-agent coalitions* considering *cross-level influences* between the data-driven themes are two mechanisms that were created to understand more in-depth how social networks and the environment influence the adoption of ORBs. These two new constructs point to a dynamic multilevel set of influences between multiple constructs (data-driven themes), developing scenarios where positive and negative health results are identified. This synthesizing argument could be considered by those designing future interventions and policy in this area, who might need to consider the wider open system of socially connected individuals and harness these forces to design new interventions where social networks and other contextual and individual factors operate together in a complex multilevel environment.

## Additional files

Additional file 1: All data-driven. (DOCX 66 kb)

Additional file 2: Coalitions. (DOCX 33 kb)

## Data Availability

All data generated or analysed during this study are included in this published article [and its supplementary information files].

## Abbreviations

CIS: Critical interpretative synthesis
LOA: Lines-of argument synthesis
LTCs: Long-term conditions
NHS: National Health System
ORBs: Obesity-related behaviours
UK: United Kingdom
USA: United States of America
