# Supplementary material for: Social network influences and the adoption of obesity-related behaviours in adults: a critical interpretative synthesis review
Source: BMC Public Health. 2019 Aug 28;19:1178. doi: 10.1186/s12889-019-7467-9 (PMC6712627; doi:10.1186/s12889-019-7467-9)
Supplement: Supplementary file 1 — All data-driven. (DOCX 66 kb) [file 12889_2019_7467_MOESM1_ESM.docx]

Additional file 1. All data-driven

**Data-driven Themes**

1. **Meso-micro network processes**

**Meso-level**

1.1 Social support

*1.1.1 Peer support*

Q1(+). “My doctors said before that I need to, to walk more … I mean it’s the kind of thing that you sort of – you kind of know anyway really within yourself that you know you’re not doing enough of any kind of exercise. This [study invitation] is what gave me the stimulus if you like to actually get on and do something about it … and even the doctor didn’t even manage to persuade me of that.” [58]

Q2(+). “Several participants suggested external health promoters could provide additional encouragement: ‘Somebody coming in from outside, say doing half an hour at lunchtime just doing a presentation about it or, you know, longer and getting people there and talking about that and saying ‘and we have our in-house person who you know if you want to talk to him, d’you wanna get encouragement from him/her’ that would be great but I think somebody coming in from outside actually would be a good idea.” [58]

*1.1.2 Group support*

T1(+). “During follow-up visits all but one woman in the study agreed that men and women were not active together. In contrast, women reported discussing exercise with other women and joining all-female exercise groups.” [47]

Q3(+). “When I had to reduce weight, I went to a weight loss group at ‘X’… When they realized that I was a cook I was engaged to take part in cooking courses and things like that for overweight people with diabetes who have challenges with their intake…” [49]

T2(-). “Participation and attendance at the pub involve negotiations and a counterbalance of the intake of beer and the health promoting effects of positive social relationships taking place at the pub.” [51]

Q4(+). “That’s why I think the group would be kind of cool to get together with… to get together as a group and just share some ideas ...” [55]

*1.2 Homophily*

T3(+). “During follow-up visits all but one woman in the study agreed that men and women were not active together. In contrast, women reported discussing exercise with other women and joining all-female exercise groups.” [47]

Q5(+). “When I had to reduce weight, I went to a weight loss group at ‘X’… When they realized that I was a cook I was engaged to take part in cooking courses and things like that for overweight people with diabetes who have challenges with their intake…” [49]

T4(+). “Most of the women agreed that they prefer walking with other women; they feel safer and more comfortable.” [52]

T5(+). “Persons of the same sex had relatively greater influence on each other than those of the opposite sex.” [68]

*1.3 Social pressure*

Q6(+). “I probably would pass somebody from my gym somewhere on the streets [… It’s] motivational in the sense that if you don’t go […to the gym] and pass a girl that I haven’t seen in a while ‘hey why I don’t see you in the gym? What’s going on with you?’ and I guess guilt people into coming back. So yeah it’s motivation.” [47]

Q7(+). “Cause for instance I sit home and I wouldn’t walk, but if somebody called me and I know I’m in a group and we walkin’ today, I’ll try to walk…cause you don’t wanna let anybody down.” [50]

Q8(+). “What happened to you the other day? Why didn’t you come walking? And it’s kind of – its sort of an accountability where if you’ve got that moral support from saying ‘What’s up? You missed two days.” [50]

T6(-). “Participation and attendance at the pub involve negotiations and a counterbalance of the intake of beer and the health promoting effects of positive social relationships taking place at the pub.” [51]

Q9(-). “However, if I go back to the village where my husband comes from, they are country people and they love to bake, cook and it’s lovely. It’s gorgeous and because they know you’re coming for afternoon tea, they’ll have made you the apple pie and they’ll have made you the cakes and if you went in there and tried to start explaining that you don’t eat any of that… In that sort of culture, it just would not be understood, and also you’re interfering with the social norms and you don’t want to do that.” [51]

T7(-). “Participants described food-centric social events as a primary constraint to eating well. Limited entertainment options in these rural communities meant that most activities involved getting together for a snack or meal. Food provision was regarded as a sign of “hospitality” and people felt obligated to eat whatever was offered in social settings (e.g., church, senior centers).” [55]

Q10(+). “An element of competition may help… if the progress was recorded and shared between all the people on the scheme, it could possibly have a positive effect…” [58]

*1.4 Natural communication*

T8(+). “WhatsApp groups comprised of women in the same exercise class could make this social pressure and social support even stronger.” [47]

*1.5 Social modelling*

Q11(+). “Participants described a program leader who would help in these areas. One expressed a desire for such a person “to motivate us or have the knowledge, but both would be good.” Another described this role as “It’s somebody that will enhance the group to walk.” [50]

Q12(+). “We have people in my neighbourhood that you can be leaving out at five in the morning, and they’re walking. You can come in at six in the afternoon and there’s another group walking…We have a monthly HOA [homeowners association] meeting— and sometimes in those meetings people just go, “Hey, I saw you walking. Can I join your group?” [53]

*1.6 Diffusion*

T9(+). “All participants received a Walk Member Handbook with community trail maps and other information sheets about issues of interest.” [50]

Q13(+). “That’s why I think the group would be kind of cool to get together with… to get together as a group and just share some ideas…” [55]

T10(+). “Much of this health information was viewed with a degree of scepticism, particularly claims made about food. In contrast to the scepticism levelled at scientific, medical and government information sources, health messages that derived from within participants’ own social network were given more credence.” [55]

Q14(+). “Overall the promoters found their booklet ‘was well set out’ and helped them approach participants: ‘It was informative and useful and helped me set out what I needed to do, promote walking to work to the colleagues, and how to approach them and stuff, I thought it was quite good.” [58]

**Micro-level**

*1.7 Social modelling*

T11(+). “When asked about recommended strategies for encouraging a friend to walk, participants emphasized social support in the form of direct encouragement (e.g., “you can make it”), serving as a role model, and offering to walk with the friend.” [50]

T12(+). “In contrast, having tight social networks was viewed as beneficial if friends were “health-conscious” and acted as positive role models.” [55]

*1.8 Social comparison*

T13(+). “While describing their motivations for pursuing physical activity, some women explained that witnessing friends and family with long term chronic conditions motivated them to take control of their own health as much as possible.” [47]

Q15(-). “I think it’s also about what I know I’m missing out on if I do exercise more. I mean, my friends and family are often going out of an evening, seeing films or going out to dinner or for drinks. I’d have to miss out on all of that if I was prioritising exercise a couple of nights a week, or even just eating really healthily would inhibit my ability to just do things like that. I don’t want to miss out.” [54]

T14(+). “Watching a family member’s health deteriorate came as a wake-up call to change their own behaviour.” [55]

Q16(+). “My grandmother… when I was 13… I was the sole witness to her coronary occlusion which killed her on the spot and I never quite dealt with that so it has left me with a bit of a fear of heart disease and heart problems and seeing how violently they can end your life.” [56]

T15(+/-). “In the case of body size, a descriptive norms effect can work through direct comparison so that a person compares himself to others in his social reference group and makes decisions regarding his own status according to that metric.” [45]

T16(+). “Relative to social comparisons to targets of the same weight, weight-focused comparisons to both thinner and heavier individuals led to increased thoughts of dieting and exercising. Moreover, comparisons to thinner targets also increased the likelihood of engaging in actual dieting and exercising behaviours. Weight comparisons to friends amplified these effects.” [66]

*1.9 Social pressure*

T17(-). “Social events involving food were areas where maintaining normal social ties were often more important than attempting to force attention on dietary needs.” [49]

Q17(+). “My husband insists that I shouldn’t eat large quantities or any starchy food. My mum always scolds me, but this doesn’t help; she just gets on my nerves. As soon as she sees me eating even the smallest amount of sweets, she’ll start complaining. I can’t say my daughters are indifferent. They’ll remark when I overeat something. Everyone is focused on my diet.” [51]

T18(-). “This man emphasizes the need to occasionally not adhere to the diet, especially at parties and when with friends.” [51]

Q18(-). “Often, when [you are] offered a piece of chocolate, you don’t say that you have diabetes, you take it and eat it just because you don’t want to offend the person and you feel uncomfortable admitting you have diabetes and you shouldn’t eat sweets.” [51]

Q19(+). “If you have someone that’s going to encourage you, say, “Come on, let’s go, let’s go do this,” or “Come on, it’s only going to take 10 minutes,” or something like that, then you go,“Okay, I’ll do it.” [53]

T19(+). “One motivator for weight-loss which was raised specifically by young adults was to feel more confident when approaching people they find attractive, and forming intimate relationships.” [54]

Q20(-). “When I go to parties or I meet up with friends, I don’t go with my diet, I just eat whatever.” [54]

T20(+/-). “Depending on these factors, regularly shared meals, such as family meals, might more or less conform to dietary guidelines although they are often generally perceived as healthy meals.” [62]

1.10 Social support

*1.10.1 Peer support*

T21(+). “Asked about who they last heard talking about physical activity, some women explained that they talked with female friends about being active regularly. For these women, peer encouragement was reported to be a strong motivator.” [47]

T22(+). “Collective efficacy was most frequently obtained through a respondent’s partner changing their lifestyle, especially in cooking and eating, to make life easier for the partner with diabetes.” [49]

Q21(+). “In the last 2 years things have worsened, because my wife got ill; she was the one who took care of me – she cooked and pleased me with delicious meals, but now she can’t anymore and I have to take care of her and of me (alone). Now, my son started helping me – he buys drugs and provides me with insulin.” [49]

Q22(+). “Another participant highlighted the instrumental role played by family members, such as a granddaughter who might say, “Come on Grandma, let’s walk. You know you’re supposed to walk. Now come on, let’s go.” [50]

Q23(+). “My wife makes cakes, but not lately as she doesn’t want to tempt me. But I know they are in the fridge… If we have a barbecue, my family serves me a Diet Coke.” [51]

T23(+). “Meals often involve family gatherings, and negotiations take place related to norms and culture of gender and family life. It appears in the interviews that female partners often play a supportive and active role monitoring their male partner’s diabetes diet underlining gender differences in diabetes.” [51]

T24(+). “However, many men attributed their healthier eating habits to their wives’ food preparation and procurement efforts (eg, home canning and gardening). Some women also discussed successful compromises during family meal times including serving smaller portions, making healthy recipe modifications, and preparing separate meals.” [55]

T25(+). “Several women cited peer support as a primary motivator for maintaining healthy eating habits.” [55]

Q24(+). It’s healthy [referring to diet] because I was brought up by my mother who was a very good cook.” [56]

Q25(+). “I used to eat a lot of vegetables when I was at home, cause my wife was an extremely good cook, so we ate really well, I don’t mean gluttony I mean just healthy food.” [56]

T26(+). “Receiving spousal/partner support also reduced mortality risk 19% (HR 0.81, 95% CI 0.66-.99).” [62]

T27(+). “Having a spouse was associated with a better physical health status (B = 1.01), especially for patients with a high income.” [18]

T28(+). “In a multivariable regression model, greater weight loss was associated with help from a child with eating goals (p=.0002) and co-worker help with physical activity (p=.01).” [64]

Q26(+). “My son would say let's walk instead of taking the train or bus.” [64]

*1.10.2 Group support*

Q27(+). “He has performed some navigation work in terms of making judgements of who he might contact in case of emergency, but does not need to do much negotiation as the support that his family provides him with in terms of diet and help in contexts where he does not feel as autonomous, supplements these needs.” [49]

Q28(+). “We are trying to exercise together, all of us… We aim to create a large group and include family and kids and socialize very often, so it becomes a big group and better habits.” [49]

Q29(+). “What works for me is walking. That’s the only thing that really works. It makes my daughter, who also is headed toward weight problems-it gives her something completely different. She’s trying to do half marathons now. So, we’re both-we see ourselves working into way more activity, even at this age. Even my husband’s getting into it… So, it’s really become a family affair kind of thing… Because that’s the only way I think we’re going to keep it going.” [53]

T29(+/-). “Family was a social influence for many, but the effect seemed highly varied. While some found criticisms from their family about being overweight helpful and motivating, others found it discouraging and hurtful.” [54]

T30(+). “In addition to reinforcing family bonds, these participants felt that exercising with their spouse or close family member provided the accountability needed to maintain good habits. For several participants, pets provided much needed companionship and reason to be active. Pets appeared to be especially important motivators of physical activity for elderly individuals living alone.” [55]

Q30(+). “[If] you wanna eat healthy… you pretty much have to change your friends at that point in time. And like in [this town], who you gonna change your friends to? Ya know, it’s not like you have nine hundred thousand other people that you can go out with and visit with.” [55]

Q31(+). “…my health…[is] my family…My children and husband, and our whanau whanui (tribal family) … our wellbeing is whanau (family)…[when] someone else is not well in our family, that has an impact…on our health…I’m connected to those people and our children…the heavier we are collectively, the better off we are individually…” [57]

T31(+). “By contrast, decreasing family contact was associated with lower scores among over-50s with infrequent friend contact such that rare/no contact showed a -1.01 unit difference (p < 0.05) compared to daily contact. Differences in friend contact of one vegetable item/day were significant (p ¼ 0.056) for adults with rare/no family contact.” [61]

T32(+). “Participants reporting family support had a 19% lower risk of mortality as compared to participants reporting no family support (P=.01).” [65]

*1.11 Homophily*

T33(+). “Asked about who they last heard talking about physical activity, some women explained that they talked with female friends about being active regularly. For these women, peer encouragement was reported to be a strong motivator.” [47]

T34(+). “Most of the women agreed that they prefer walking with other women; they feel safer and more comfortable.” [52]

T35(+). “More precisely, women and their most important eating companions tended to be similar in diet-related factors such as diet quality and eating styles as well as in BMI.” [62]

T36(-). “These results indicated that an unhealthy social eating environment might be a risk factor for the development of unhealthy eating patterns and obesity.” [62]

T37(+). “The results of this study show that the probability of engaging in regular exercise or eating a healthy diet is higher when individuals have friends who also engage in these behaviours.” [63]

Q32(+). “What works for me is walking. That’s the only thing that really works. It makes my daughter, who also is headed toward weight problems-it gives her something completely different. She’s trying to do half marathons now. So, we’re both-we see ourselves working into way more activity, even at this age. Even my husband’s getting into it… So, it’s really become a family affair kind of thing… Because that’s the only way I think we’re going to keep it going.” [53]

T38(+). “During treatment, participants lost an average of 4.4% of initial body weight, and social influence factors were adversely associated with weight loss outcomes. Having more casual friends who were overweight at baseline and being part of a social network with stronger social norms for unhealthy eating predicted poorer weight losses (p’s<.023).” [27]

T39(+). “Persons of the same sex had relatively greater influence on each other than those of the opposite sex.” [68]

T40(+). “The sex of the ego and alter also appeared to be important. When the sample was restricted to same-sex friendships (87% of the total), the probability of obesity in an ego increased by 71% (95% CI, 13 to 145) if the alter became obese.” [68]

*1.12 Natural communication*

Q33(+). “…found myself you know doing the walking home without having written it down and you know having told several people – I mean telling people that that’s what you’re doing actually makes you hold to it even more than if you, if I’d written it down.” [58]

*1.13 Isolation*

Q34(+). “However, you get fed up and tired; so, when I have the opportunity to be alone and take it easy, I relax and do things I like to do. I also feel that I have better control of the diabetes. No outside negative influence.” [51]

T41(-). “Several elderly women also discussed the negative consequences of living alone on their diets. Without family members around, eating decisions were primarily based on convenience and several participants reported having no incentive to make dietary improvements at their advanced age.” [55]

T42(-). “We found that being single or widowed was associated with a lower variety score, particularly vegetable variety, and associations were enhanced when combined with male gender, living alone or infrequent friend contact.” [61]

1. **Contextual and individual factors**

**Meso-level**

2.1 Environmental factors

*2.1.1 Built environment*

T43(+). “While opportunities for active commuting seemed limited, interviews and participant observation revealed that most women could walk in their neighbourhoods or easily access spaces to walk.” [47]

Q35(-). “Close to my house, there are no sidewalks. And I feel like I don’t want to get in the car and drive somewhere and get out and walk and get back in the car… I used to walk a lot, but I lived somewhere else so it made it very simple.” [50]

Q36(-). ‘‘Changing diet alone without exercise will not help lose weight and there are no places to do Exercise.’’ [52]

Q37(-). Women who did not have access to a nearby physical activity resource at work or home described this as a barrier to doing physical activity. One 45-year-old African American woman states, “… If I could just walk outside, I think that’d be more easier… Ain’t got to worry about having enough gas to drive.” [53]

Q38(-). “It’s a fast food society – you don’t get many vegetables in a Big Mac.” [56]

*2.1.2 Community resources*

Q39(+). “I’m looking for more, all the time… I’m getting ideas at the moment because when I go to the sports centre they’ve got loads of activities for older people like me and other illnesses, not just diabetes, they cover everything there.” [49]

Q40(+). “When I had to reduce weight, I went to a weight loss group at ‘X’… When they realized that I was a cook I was engaged to take part in cooking courses and things like that for overweight people with diabetes who have challenges with their intake…” [49]

T44(+). “Similar to Bulgaria, in Greece (G), financial austerity led to stronger dependence on family support, but unlike Bulgaria, most people are co-located within a domestic environment. For those without family networks, the health centres played a strong supporting role.” [49]

T45(+). “In Greece, due to the financial crisis, charitable organizations are providing more of such care to the homeless.” [49]

Q41(+). ‘‘We want encouragement from the club, they should organise programmes and different sport competitions.” [52]

T46(+). “In these small rural towns, social interaction appeared to be an important facilitator of active lifestyles, particularly for women. Organized group activities, such as walking, were viewed as an opportunity to socialize with friends and connect with the community. Building these networks increased enjoyment and gave people more incentive to engage in activity.” [55]

*2.1.3 Weather*

Q42(-). “Sometimes the walk is be good you know exercise but if I have my car I wouldn’t walk at all only when I don’t have do I walk cause everything closer in town [Bridgetown] that ya could walk to instead of wasting the gas but as for out here [St. Philip]… the closest shop there … nah… now that is daytime no way! Ain’t walking. Too hot!” [47]

Q43(-). ‘‘When the weather is cold I walk, but it is difficult to walk in summer.’’ [52]

2.2 Socio-cultural factors

*2.2.1 Social events*

T47(-). “Participants described food-centric social events as a primary constraint to eating well. Limited entertainment options in these rural communities meant that most activities involved getting together for a snack or meal. Food provision was regarded as a sign of “hospitality” and people felt obligated to eat whatever was offered in social settings (e.g., church, senior centers).” [55]

2.2.2 Social norms

T48(+). “During follow-up visits all but one woman in the study agreed that men and women were not active together. In contrast, women reported discussing exercise with other women and joining all-female exercise groups.” [47]

Q44(+/-). “You know with diabetics especially with Africans to be frank Africans they don’t like saying what they are suffering from, everybody’s just hiding what is. A lot of my friends don’t know I’m diabetic, and I’ve been with them for 10 years they don’t know.” [49]

T49(-). “Participation and attendance at the pub involve negotiations and a counterbalance of the intake of beer and the health promoting effects of positive social relationships taking place at the pub.” [51]

Q45(-). “However, if I go back to the village where my husband comes from, they are country people and they love to bake, cook and it’s lovely. It’s gorgeous and because they know you’re coming for afternoon tea, they’ll have made you the apple pie and they’ll have made you the cakes and if you went in there and tried to start explaining that you don’t eat any of that… In that sort of culture, it just would not be understood, and also you’re interfering with the social norms and you don’t want to do that.” [51]

Q46(+). “Religious celebration and fasting have an impact and produce challenges for patients with diabetes. Religious factors were apparent in negotiations of food choice, especially among respondents from Greece: I’m fasting these days. I find it’s good for me. I avoid milk, meat, eggs and cheese.” [51]

Q47(-). “Walking is not culturally acceptable. My husband will not allow me to walk in the street but if it is a closed place [gym] he has no problem.” [52]

T50(+). “Most of the women agreed that they prefer walking with other women; they feel safer and more comfortable.” [52]

Q48(+). “… You can throw all the medicines and all the gym memberships [at me]… but if they don’t understand their wairautanga (spirituality)… you’ll never be that full person that our tupuna (ancestors) use to be…” [57]

**Micro-level**

2.3 Environmental factor

*2.3.1 Group atmosphere*

Q49(+). “Going to the gym the motivation is, well obviously it would be generally to lose weight, but going by the gym is relatively small so you know everybody that is there so it’s kind of a family type atmosphere.” [47]

2.4 Psychosocial factors

*2.4.1 Critical moments*

T51(+). “While describing their motivations for pursuing physical activity, some women explained that witnessing friends and family with long term chronic conditions motivated them to take control of their own health as much as possible.” [47]

Q50(+). “In the last 2 years things have worsened, because my wife got ill; she was the one who took care of me – she cooked and pleased me with delicious meals, but now she can’t anymore and I have to take care of her and of me (alone). Now, my son started helping me – he buys drugs and provides me with insulin.” [49]

T52(+). “Family health concerns were another driver of participant’s eating behaviours. Having a spouse or child diagnosed with a chronic condition motivated many people to make supportive dietary changes.” [55]

T53(+). “Watching a family member’s health deteriorate came as a wake-up call to change their own behaviour.” [55]

Q51(+). “My grandmother… when I was 13… I was the sole witness to her coronary occlusion which killed her on the spot and I never quite dealt with that so it has left me with a bit of a fear of heart disease and heart problems and seeing how violently they can end your life.” [56]

Q52(-). “There was a period going back 9 or 10 years after my mother died. . . when I was going through a very very difficult period I was living for a period off bread and porridge which was very unhealthy.” [56]

*2.4.2 Motivation*

T54(+). “Asked about who they last heard talking about physical activity, some women explained that they talked with female friends about being active regularly. For these women, peer encouragement was reported to be a strong motivator.” [47]

Q53(+). “I probably would pass somebody from my gym somewhere on the streets [… It’s] motivational in the sense that if you don’t go […to the gym] and pass a girl that I haven’t seen in a while ‘hey why I don’t see you in the gym? What’s going on with you?’ and I guess guilt people into coming back. So yeah it’s motivation.” [47]

Q54(+). “I’m looking for more, all the time… I’m getting ideas at the moment because when I go to the sports centre they’ve got loads of activities for older people like me and other illnesses, not just diabetes, they cover everything there.” [49]

Q55(-). “Sometimes I feel bored and depressed, when I try to reduce weight by eating less and exercise I see that there is no change in my weight so I feel depressed and I will stop everything and spend most of my time using the computer and internet especially after I finished university I noticed my weight increased more.” [52]

Q56(+). “There is some type of apprehension in the back of my mind, and I’m trying to figure out why, but I really need to say, “Go ahead, start doing it.” I guess I feel that if I start, I’m going to have to continue. It’s going to change my routine. [And that] Moves me out of my comfort zone.” [53]

T55(+). “One motivator for weight-loss which was raised specifically by young adults was to feel more confident when approaching people they find attractive, and forming intimate relationships.” [54]

Q57(+). “Offering a cash incentive for people to walk rather than drive.” [58]

*2.4.3 Self-efficacy*

T56(+). “Health promotion methods: guided practice, enactment, verbal persuasion, goal setting, planned coping responses.” [50]

Q58(-). “I have the control to change things I just don’t change them, and I don’t know why. It’s ridiculous.” [54]

Q59(+). “I was never wanting to do more than walk halfway to work … that was my ultimate, and that for me would be success and he [promoter] kept giving me routes and to get here and I kept saying but I’m not doing that am I because that isn’t my goal and you need to support me with my goal rather than yours … if I set my goal on what he wanted, I wouldn’t have achieved it and then I would’ve failed so I wanted to be able to succeed in what I wanted to do.” [58]

Q60(+). “I thought the apps and all the stuff that you do on the computer to be able to track and log everything and find the easier routes to walk and things like that was really, really good … I think they should be publicised a bit more because I definitely think that would help people and convince more people to walk.” [58]

T57(+). “Specifically, people who reported good self-management skills were more likely to have a diverse network, to be older, to be in relatively good health, to have high levels of income and education, and to live in the wealthier of the six countries (Norway, UK, Netherlands, Spain). High levels of self-monitoring were also associated with high education and relatively good health.” [59]

*2.4.4 Knowledge*

T58(+). “All participants received a Walk Member Handbook with community trail maps and other information sheets about issues of interest.” [50]

Q61(-). “The food we eat is not healthy because of the way we cook it and because we do not know enough about healthy food.” [52]

Q62(+/-). “[Health messages] shouldn’t be too clever or too scientific because you soon get bored with that. People understand that they should be interested, just simple messages, large clear simple messages.” [56]

Q63(+). “Overall the promoters found their booklet ‘was well set out’ and helped them approach participants: ‘It was informative and useful and helped me set out what I needed to do, promote walking to work to the colleagues, and how to approach them and stuff, I thought it was quite good.” [58]

*2.4.5 Personal attitude*

Q64(-). “I do a lot of things that jeopardize the health but to me, regardless if you do or don’t do, things still happen […] Sometimes you will see a person don’t smoke don’t drink is still see them develop well everybody got diabetes so yeah but to me it don’t matter what you do or don’t to bring it on. Things just happen…” [47]

T59(+). “Whatsapp groups comprised of women in the same exercise class could make this social pressure and social support even stronger.” [47]

T60(+). “Some women described being active at home and doing a range of activities from Zumba® DVDs to light weight lifting and floor exercises to walking on a treadmill.” [47]

Q65(-). “I feel heavy [fat] but I do not want to reduce my weight.” [52]

Q66(-). ‘‘For me, computer and Internet; I spend a lot of time using it and so there is no time for exercise.” [52]

T61(+/-). “Individuals agreed that there was a general social pressure to lose weight and be thin, particularly from the media.” [54]

Q67(-). “I think people [study participants] have the intentions of walking … but, because their character is, just they don’t know how to live without the car.” [58]

Q68(+). “I thought the apps and all the stuff that you do on the computer to be able to track and log everything and find the easier routes to walk and things like that was really, really good … I think they should be publicised a bit more because I definitely think that would help people and convince more people to walk.” [58]

T62(+/-). “Normative comparison, therefore, involves self-judgment, and, within that context, people may judge themselves to be either superior or inferior to others.” [45]

2.5 Socio-cultural factors

*2.5.1 Social norms*

T63(+). “Asked about who they last heard talking about physical activity, some women explained that they talked with female friends about being active regularly. For these women, peer encouragement was reported to be a strong motivator.” [47]

T64(-). “In some cases, the family did not provide support when it might have been expected, there may be a gender factor here – older women often did not get the support they expected from their family whereas men did (especially when widowed or divorced).” [49]

T65(+/-). “Depending on these factors, regularly shared meals, such as family meals, might more or less conform to dietary guidelines although they are often generally perceived as healthy meals.” [62]

T66(+/-). “… body size, a descriptive norms effect can work through direct comparison so that a person compares himself to others in his social reference group and makes decisions regarding his own status according to that metric.” [45]

T67(-). “During treatment, participants lost an average of 4.4% of initial body weight, and social influence factors were adversely associated with weight loss outcomes. Having more casual friends who were overweight at baseline and being part of a social network with stronger social norms for unhealthy eating predicted poorer weight losses (p’s<.023).” [27]

*2.5.2 Competing demands*

T68(-). “Several women described child rearing responsibilities as one of the primary reasons why they were not active.” [47]

Q69(-). “I have three children, a husband who works three shifts… it’s just too easy to say ‘now, you have to start exercising; exercise every day!’ So you commute 20-miles each day, and you have three kids, school, day care, a man…” [49]

Q70(-). “My son has football practice so after work it’s like I don’t have no time, just runnin’ to go get him. But I should drop him off and go walk, but once I get home he’s hungry. Everything takes over.” [50]

Q71(-). “I look after my husband, the house, everything. I don’t look after myself as much as I used to. In the past, I would cook something for myself and something for the others to eat… I have to cook meals that my children and grandchildren like because my daughter works, and so I eat from these as well, so I don’t miss out.” [51]

Q72(-). “There is no time because there is a lot of housework’’. ‘‘Sometimes appetite, we can’t prevent ourselves from eating; sometimes children are at home so no time to exercise.” [52]

T69(-). “In general, most women cited a lack of time for doing physical activity and lacked motivation and confidence if or when time became available.” [53]

Q73(-). “I really do believe that for me, and for a lot of people who are Hispanic and Black, it’s education. The parents and the grandparents not teaching us as a young child… They were either cleaning, cooking, or working. And [physical activity] that wasn’t the priority…” [53]

Q74(-). “My work and my kids take up time, which technically means that I have less time to exercise because of them, but that’s certainly not their fault… but every parent accepts that’s just part of the deal I think, so I’m okay with that.” [54]

T70(-). “Family obligations emerged as a common barrier to being physically active. Women in these rural households frequently assumed traditional caregiving roles and prioritized time with their kids and husbands over exercise. Some articulated concerns over abandoning family responsibilities to fulfill their own needs.” [55]

Q75(-). “Um at the moment [my diet is] wickedly poor, very poor cause I’m busy at the moment, I don’t really have time to cook.” [56]

Q76(-). “They’ve got to pick up children from schools or clubs or some people have elderly relatives that they look after… some people will be rushing home to go off to yoga, pilates, dance so it depends if it’s going to take a lot longer.” [58]

*2.5.3 Social events*

T71(-). “Social events involving food were areas where maintaining normal social ties were often more important than attempting to force attention on dietary needs.” [49]

Q77(-). “When I go to parties or I meet up with friends, I don’t go with my diet, I just eat whatever.” [54]

T72(-). “This man emphasizes the need to occasionally not adhere to the diet, especially at parties and when with friends.” [51]

Q78(-). “I’ve got cakes in the house and everything else and I’ve got them all in for Christmas, I mean it’s for everybody else. So, it’s neither here nor there. I think they just all accept that I won’t eat them and that’s it, and they keep telling me I’m too strong willed.” [51]

2.6 Sociodemographic factors

*2.6.1 Socio-economic status*

Q79(-). “I would like to find a gym that is cheap that I could get a personal trainer. Ain’t cheap! Man, tell me is 60 dollars a session! That is 60 dollars for one session one 45 minute session! Not even an hour.” [47]

Q80(-). “Without the help of my children, I wouldn’t be able to cope. My pension is 140 leva—[not enough] for following a diet and buying drugs.” [51]

Q81(-). “Budget. Like not everyone can afford a gym membership. I mean they’re pretty absurd.” [54]

T73(-). “Both “male” and “low-income status” are significant risk factors for eating an inappropriate diet.” [56]

T74(+/-). “People in Family supported networks were more likely to be women, and were characterised by lower education and income levels amongst ‘egos’, but had high levels of network member involvement and reported high levels of well-being.” [59]

T75(+). “Specifically, people who reported good self-management skills were more likely to have a diverse network, to be older, to be in relatively good health, to have high levels of income and education, and to live in the wealthier of the six countries (Norway, UK, Netherlands, Spain). High levels of self-monitoring were also associated with high education and relatively good health.” [59]

T76(+). “Having a spouse was associated with a better physical health status (B = 1.01), especially for patients with a high income.” [18]

T77(+). “Attending community organizations was positively related to physical activity, however only for patients with a low income (OR = 1.53).” [18]

*2.6.2 Job*

Q82(-). “I worked as […] a cashier at a supermarket until 2009 and you know a cashier sits down ain’t much activity in that and then in 2009 to 2011 I did secretarial work – so that’s even worse! Cause […] when you’re sitting down and working all day sitting down Facebook – you know after you ya know finish your work – but then […] I got this new job that I totally love cause since I really can’t get the exercise that I want to put in, I think it give me a little moderate […] up and down.” [47]

*2.6.3 Age*

T78(-). “Some young adults reported that the media provided motivation to lose weight. In contrast, older adults described actively attempting to resist it through the way they engage with and respond to such pressure.” [54]

T79(+). “Specifically, people who reported good self-management skills were more likely to have a diverse network, to be older, to be in relatively good health, to have high levels of income and education, and to live in the wealthier of the six countries (Norway, UK, Netherlands, Spain). High levels of self-monitoring were also associated with high education and relatively good health.” [59]

*2.6.4 Gender*

T80(+/-). “Young females spoke about wanting to lose weight and reduce body fat, while males described intentions to build muscle mass and ‘bulk up.” [54]

T81(+). “In these small rural towns, social interaction appeared to be an important facilitator of active lifestyles, particularly for women. Organized group activities, such as walking, were viewed as an opportunity to socialize with friends and connect with the community. Building these networks increased enjoyment and gave people more incentive to engage in activity.” [55]

T82(-). “In contrast, men were less likely to view structured group exercise as a social activity.” [55]

T83(-). “Both “male” and “low-income status” are significant risk factors for eating an inappropriate diet.” [56]

T84(+). “People in Family supported networks were more likely to be women, and were characterised by lower education and income levels amongst ‘egos’, but had high levels of network member involvement and reported high levels of well-being.” [59]

T85(-). “We found that being single or widowed was associated with a lower variety score, particularly vegetable variety, and associations were enhanced when combined with male gender, living alone or infrequent friend contact.” [61]

T86(-). “Negative associations between loneliving and variety scores were also stronger in men: lone-living men had a -1.46 unit difference (p < 0.001) in vegetable variety score which was significantly different (p ¼ 0.001) from the -0.66 unit difference (p < 0.001) in score for lone-living women, compared to co-living counterparts.” [61]

T87(+). “The sex of the ego and alter also appeared to be important. When the sample was restricted to same-sex friendships (87% of the total), the probability of obesity in an ego increased by 71% (95% CI, 13 to 145) if the alter became obese.” [68]

T88(+). “Persons of the same sex had relatively greater influence on each other than those of the opposite sex.” [68]

*2.6.5 Transport*

Q83(-). “Sometimes the walk is be good you know exercise but if I have my car I wouldn’t walk at all only when I don’t have do I walk cause everything closer in town [Bridgetown] that ya could walk to instead of wasting the gas but as for out here [St. Philip]… the closest shop there … nah… now that is daytime no way! Ain’t walking. Too hot!” [47]

Q84(-). “I think people [study participants] have the intentions of walking … but, because their character is, just they don’t know how to live without the car.” [58]

*2.6.6 Education*

Q85(-). “I really do believe that for me, and for a lot of people who are Hispanic and Black, it’s education. The parents and the grandparents not teaching us as a young child… They were either cleaning, cooking, or working. And [physical activity] that wasn’t the priority…” [53]

T89(-). “People in Family supported networks were more likely to be women, and were characterised by lower education and income levels amongst ‘egos’, but had high levels of network member involvement and reported high levels of well-being.” [59]

T90(+). “Specifically, people who reported good self-management skills were more likely to have a diverse network, to be older, to be in relatively good health, to have high levels of income and education, and to live in the wealthier of the six countries (Norway, UK, Netherlands, Spain). High levels of self-monitoring were also associated with high education and relatively good health.” [59]

2.7 Clinical factors

*2.7.1 Medical condition*

Q86(-). “I cannot move a lot because I had a stroke and so I cannot walk or do any exercise and this is why I gained weight.’’ [52]

T91(+/-). “People with type 2 diabetes were less physically active, less likely to follow recommended diet (men), had fewer contacts with family and friends and were less certain of counting on help in case of severe illness than people with type 1 diabetes.” [67]

1. **Types of ties and properties of social networks**

**Meso-level (weak ties)**

*3.1 Sport contacts*

T92(+). “During follow-up visits all but one woman in the study agreed that men and women were not active together. In contrast, women reported discussing exercise with other women and joining all-female exercise groups.” [47]

T93(+). “Asked about who they last heard talking about physical activity, some women explained that they talked with female friends about being active regularly. For these women, peer encouragement was reported to be a strong motivator.” [47]

Q87(+). “I probably would pass somebody from my gym somewhere on the streets [… It’s] motivational in the sense that if you don’t go […to the gym] and pass a girl that I haven’t seen in a while ‘hey why I don’t see you in the gym? What’s going on with you?’ and I guess guilt people into coming back. So yeah it’s motivation.” [47]

T94(+). “WhatsApp groups comprised of women in the same exercise class could make this social pressure and social support even stronger.” [47]

*3.2 Healthcare professionals*

T95(+). “In Bulgaria compared to elsewhere, health professionals’ advice was taken more seriously and sought more frequently.” [49]

Q88(+). “We want a dietitian in each club to help us lose weight.” [52]

Q89(+). “My doctors said before that I need to, to walk more … I mean it’s the kind of thing that you sort of – you kind of know anyway really within yourself that you know you’re not doing enough of any kind of exercise. This [study invitation] is what gave me the stimulus if you like to actually get on and do something about it … and even the doctor didn’t even manage to persuade me of that.” [58]

Q90(-). “…They [doctors] all say I’m too heavy…they give you medication but it obviously hasn’t worked…I’m just not used to these medications…” [59]

*3.3 Neighbours*

Q91(+). “We have people in my neighbourhood that you can be leaving out at five in the morning, and they’re walking. You can come in at six in the afternoon and there’s another group walking… We have a monthly HOA [homeowners association] meeting— and sometimes in those meetings people just go, “Hey, I saw you walking. Can I join your group?” [53]

*3.4 Community organizations and community (others)*

T96(+). “In Greece, due to the financial crisis, charitable organizations are providing more of such care to the homeless.” [49]

Q92(+). “When I had to reduce weight, I went to a weight loss group at ‘X’… When they realized that I was a cook I was engaged to take part in cooking courses and things like that for overweight people with diabetes who have challenges with their intake…” [49]

Q93(+). “Cause for instance I sit home and I wouldn’t walk, but if somebody called me and I know I’m in a group and we walkin’ today, I’ll try to walk…cause you don’t wanna let anybody down.” [50]

Q94(+). “What happened to you the other day? Why didn’t you come walking? And it’s kind of – its sort of an accountability where if you’ve got that moral support from saying ‘What’s up? You missed two days.” [50]

Q95(-). “However, if I go back to the village where my husband comes from, they are country people and they love to bake, cook and it’s lovely. It’s gorgeous and because they know you’re coming for afternoon tea, they’ll have made you the apple pie and they’ll have made you the cakes and if you went in there and tried to start explaining that you don’t eat any of that… In that sort of culture, it just would not be understood, and also you’re interfering with the social norms and you don’t want to do that.” [51]

T97(+). “In these small rural towns, social interaction appeared to be an important facilitator of active lifestyles, particularly for women. Organized group activities, such as walking, were viewed as an opportunity to socialize with friends and connect with the community. Building these networks increased enjoyment and gave people more incentive to engage in activity.” [55]

T98(-). “Participants described food-centric social events as a primary constraint to eating well. Limited entertainment options in these rural communities meant that most activities involved getting together for a snack or meal. Food provision was regarded as a sign of “hospitality” and people felt obligated to eat whatever was offered in social settings (eg, church, senior centers).” [55]

Q96(+). “That’s why I think the group would be kind of cool to get together with… to get together as a group and just share some ideas ...” [55]

Q97(-). “I was never wanting to do more than walk halfway to work … that was my ultimate, and that for me would be success and he [promoter] kept giving me routes and to get here and I kept saying but I’m not doing that am I because that isn’t my goal and you need to support me with my goal rather than yours … if I set my goal on what he wanted, I wouldn’t have achieved it and then I would’ve failed so I wanted to be able to succeed in what I wanted to do.” [58]

Q98(+). “Several participants suggested external health promoters could provide additional encouragement: ‘Somebody coming in from outside, say doing half an hour at lunchtime just doing a presentation about it or, you know, longer and getting people there and talking about that and saying ‘and we have our in-house person who you know if you want to talk to him, d’you wanna get encouragement from him/her’ that would be great but I think somebody coming in from outside actually would be a good idea.” [58]

T99(+). “Attending community organizations was positively related to physical activity, however only for patients with a low income (OR = 1.53).” [18]

**Micro-level (close bond connections)**

*3.5 Family*

Q99(-). “Some women don’t have help - the children have dads that don’t help or other family members so that’s why too.” [47]

T100(+). “While describing their motivations for pursuing physical activity, some women explained that witnessing friends and family with long term chronic conditions motivated them to take control of their own health as much as possible.” [47]

T101(-). “In some cases, the family did not provide support when it might have been expected, there may be a gender factor here – older women often did not get the support they expected from their family whereas men did (especially when widowed or divorced).” [49]

Q100(+). “We are trying to exercise together, all of us… We aim to create a large group and include family and kids and socialize very often, so it becomes a big group and better habits.” [49]

Q101(+). “My children have booked me a private doctor. I don’t know how much it costs – they won’t tell me. And if I haven’t gone to the private clinic for a long time, they call me… My family takes my illness into consideration, they even monitor me. When I go to visit them my children make efforts to cook diet food.” [49]

Q102(-). “No, my family doesn’t help me. I am responsible for health issues at home… I ask them to support me a bit more, taking the cakes out of my sight, but they’re all tomboy-like and take little care of me. They don’t see a disease in my diabetes, I’m telling you, they don’t see it at all… So in that sense I don’t have anybody, anybody that tells me: ‘hey, you’re exceeding yourself, don’t eat that’.” [49]

T102(+). “Collective efficacy was most frequently obtained through a respondent’s partner changing their lifestyle, especially in cooking and eating, to make life easier for the partner with diabetes.” [49]

Q103(+). “In the last 2 years things have worsened, because my wife got ill; she was the one who took care of me – she cooked and pleased me with delicious meals, but now she can’t anymore and I have to take care of her and of me (alone). Now, my son started helping me – he buys drugs and provides me with insulin.” [49]

Q104(+). “He has performed some navigation work in terms of making judgements of who he might contact in case of emergency, but does not need to do much negotiation as the support that his family provides him with in terms of diet and help in contexts where he does not feel as autonomous, supplements these needs.” [49]

Q105(+). “Another participant highlighted the instrumental role played by family members, such as a granddaughter who might say, “Come on Grandma, let’s walk. You know you’re supposed to walk. Now come on, let’s go.” [50]

T103(+). “When asked about recommended strategies for encouraging a friend to walk, participants emphasized social support in the form of direct encouragement (e.g., “you can make it”), serving as a role model, and offering to walk with the friend.” [50]

T104(+/-). “This perspective relates to how diet affects close family relations and accordingly how the individual with diabetes necessarily involves family members in customary food practices. The changes obviously involve abandoning habits that family members were used to and enjoyed.” [51]

Q106(+). “My wife makes cakes, but not lately as she doesn’t want to tempt me. But I know they are in the fridge… If we have a barbecue, my family serves me a Diet Coke.” [51]

Q107(-). “To my regret, my husband doesn’t have pity on me, or understand my illness and he even takes my diabetes quite light-heartedly. He doesn’t give me money for medicines or for buying better quality foods, so that I can stick to a healthy lifestyle and keep up a proper diet for this type 2 diabetes.” [51]

Q108(+). “Without the help of my children, I wouldn’t be able to cope. My pension is 140 leva—[not enough] for following a diet and buying drugs.” [51]

T105(+). “Meals often involve family gatherings, and negotiations take place related to norms and culture of gender and family life. It appears in the interviews that female partners often play a supportive and active role monitoring their male partner’s diabetes diet underlining gender differences in diabetes.” [51]

Q109(+). “My husband insists that I shouldn’t eat large quantities or any starchy food. My mum always scolds me, but this doesn’t help; she just gets on my nerves. As soon as she sees me eating even the smallest amount of sweets, she’ll start complaining. I can’t say my daughters are indifferent. They’ll remark when I overeat something. Everyone is focused on my diet.” [51]

Q110(-). “Walking is not culturally acceptable. My husband will not allow me to walk in the street but if it is a closed place [gym] he has no problem.” [52]

Q111(-). “My family won’t encourage me; for example if I want to bring a walking machine they say there is no place to keep it.” [52]

Q112(-). “I really do believe that for me, and for a lot of people who are Hispanic and Black, it’s education. The parents and the grandparents not teaching us as a young child… They were either cleaning, cooking, or working. And [physical activity] that wasn’t the priority…” [53]

Q113(+). “What works for me is walking. That’s the only thing that really works. It makes my daughter, who also is headed toward weight problems-it gives her something completely different. She’s trying to do half marathons now. So, we’re both-we see ourselves working into way more activity, even at this age. Even my husband’s getting into it… So, it’s really become a family affair kind of thing… Because that’s the only way I think we’re going to keep it going.” [53]

Q114(-). “I think it’s also about what I know I’m missing out on if I do exercise more. I mean, my friends and family are often going out of an evening, seeing films or going out to dinner or for drinks. I’d have to miss out on all of that if I was prioritising exercise a couple of nights a week, or even just eating really healthily would inhibit my ability to just do things like that. I don’t want to miss out.” [54]

T106(+/-). “Family was a social influence for many, but the effect seemed highly varied. While some found criticisms from their family about being overweight helpful and motivating, others found it discouraging and hurtful.” [54]

Q115(+/-). “I eat what my mum gives me. Usually my mum makes all of my dinners and gives me a packed lunch, so my diet is pretty much all that I need.” [54]

T107(+). “Food choice negotiations within the family emerged as a key determinant of eating behaviour. As the primary food preparers, women frequently reported difficulties in reconciling their spouses’ food preferences with their own desires to eat healthfully.” [55]

T108(+). “However, many men attributed their healthier eating habits to their wives’ food preparation and procurement efforts (e.g., home canning and gardening). Some women also discussed successful compromises during family meal times including serving smaller portions, making healthy recipe modifications, and preparing separate meals.” [55]

T109(+). “Family health concerns were another driver of participant’s eating behaviours. Having a spouse or child diagnosed with a chronic condition motivated many people to make supportive dietary changes.” [55]

T110(+). “Watching a family member’s health deteriorate came as a wake-up call to change their own behaviour.” [55]

T111(+). “In addition to reinforcing family bonds, these participants felt that exercising with their spouse or close family member provided the accountability needed to maintain good habits. For several participants, pets provided much needed companionship and reason to be active. Pets appeared to be especially important motivators of physical activity for elderly individuals living alone.” [55]

Q116(+). “My grandmother… when I was 13… I was the sole witness to her coronary occlusion which killed her on the spot and I never quite dealt with that so it has left me with a bit of a fear of heart disease and heart problems and seeing how violently they can end your life.” [56]

Q117(+). “I used to eat a lot of vegetables when I was at home, cause my wife was an extremely good cook, so we ate really well, I don’t mean gluttony I mean just healthy food.” [56]

Q118(+). “It’s healthy [referring to diet] because I was brought up by my mother who was a very good cook.” [56]

Q119(+). “…my health…[is] my family…My children and husband, and our whanau whanui (tribal family) … our wellbeing is whanau (family)…[when] someone else is not well in our family, that has an impact…on our health…I’m connected to those people and our children…the heavier we are collectively, the better off we are individually…” [57]

T112(+/-). “Depending on these factors, regularly shared meals, such as family meals, might more or less conform to dietary guidelines although they are often generally perceived as healthy meals.” [66]

T113(+). “By contrast, decreasing family contact was associated with lower scores among over-50s with infrequent friend contact such that rare/no contact showed a -1.01 unit difference (p < 0.05) compared to daily contact. Differences in friend contact of one vegetable item/day were significant (p ¼ 0.056) for adults with rare/no family contact.” [61]

T114(+). “In a multivariable regression model, greater weight loss was associated with help from a child with eating goals (p=.0002) and co-worker help with physical activity (p=.01).” [64]

T115(-). “Weight gain was associated with having network members with obesity living in the home (p=.048) and increased network size (p=.002).” [64]

Q120(+). “My son would say let's walk instead of taking the train or bus.” [64]

Q121(-). “My husband and sister criticized me. If they saw me serve less food or eat vegetables they said I wanted to be sexy. They made fun of me because I was eating healthier than before. They said I just wanted to be skinny.” [64]

T116(+). “Participants reporting family support had a 19% lower risk of mortality as compared to participants reporting no family support (P=.01).” [65]

T117(+). “Receiving spousal/partner support also reduced mortality risk 19% (HR 0.81, 95% CI 0.66-.99).” [55]

T118(+). “Having a spouse was associated with a better physical health status (B = 1.01), especially for patients with a high income.” [18]

*3.6 Friends*

T119(+). “While describing their motivations for pursuing physical activity, some women explained that witnessing friends and family with long term chronic conditions motivated them to take control of their own health as much as possible.” [47]

T120(-). “Social events involving food were areas where maintaining normal social ties were often more important than attempting to force attention on dietary needs.” [49]

Q122(+/-). “You know with diabetics especially with Africans to be frank Africans they don’t like saying what they are suffering from, everybody’s just hiding what is. A lot of my friends don’t know I’m diabetic, and I’ve been with them for 10 years they don’t know.” [49]

T121(-). “This man emphasizes the need to occasionally not adhere to the diet, especially at parties and when with friends.” [51]

T122(-). “Participation and attendance at the pub involve negotiations and a counterbalance of the intake of beer and the health promoting effects of positive social relationships taking place at the pub.” [51]

T123(+). “One motivator for weight-loss which was raised specifically by young adults was to feel more confident when approaching people they find attractive, and forming intimate relationships.” [54]

Q122(-). “I think it’s also about what I know I’m missing out on if I do exercise more. I mean, my friends and family are often going out of an evening, seeing films or going out to dinner or for drinks. I’d have to miss out on all of that if I was prioritising exercise a couple of nights a week, or even just eating really healthily would inhibit my ability to just do things like that. I don’t want to miss out.” [54]

Q123(-). “When I go to parties or I meet up with friends, I don’t go with my diet, I just eat whatever.” [54]

T124(+). “In contrast, having tight social networks was viewed as beneficial if friends were “health-conscious” and acted as positive role models.” [55]

T125(+). “In these small rural towns, social interaction appeared to be an important facilitator of active lifestyles, particularly for women. Organized group activities, such as walking, were viewed as an opportunity to socialize with friends and connect with the community. Building these networks increased enjoyment and gave people more incentive to engage in activity.” [55]

Q124(-). “[If] you wanna eat healthy… you pretty much have to change your friends at that point in time. And like in [this town], who you gonna change your friends to? Ya know, it’s not like you have nine hundred thousand other people that you can go out with and visit with.” [55]

T126(+). “Moreover, having more friends is associated with an improvement in health, while being healthy and prosocial is associated with closer relationships. Specifically, a unit increase in health is associated with an expected 0.45 percentage-point increase in average closeness, while adding a prosocial activity is associated with a 0.46 percentage-point increase in the closeness of one’s relationships.” [60]

T127(+). “In response to the name generators (‘‘Who do you spend free time with’’ and ‘‘Who do you discuss important issues with’’), we found that Americans identify an average of 4.4+-1.8 close social contacts (the average respondent lists 2.2 friends, 0.76 spouses, 0.28 siblings, 0.44 co-workers, and 0.30 neighbours).” [60]

T128(+). “The results of this study show that the probability of engaging in regular exercise or eating a healthy diet is higher when individuals have friends who also engage in these behaviours.” [63]

T129(+). “Participants reporting social contact with 6 or 7 friends on a weekly basis had a 24% lower mortality risk than those in contact with ≤ 1 friend (HR 0.76, 95% CI 0.58–0.98).” [65]

T130(+/-). “Relative to social comparisons to targets of the same weight, weight-focused comparisons to both thinner and heavier individuals led to increased thoughts of dieting and exercising. Moreover, comparisons to thinner targets also increased the likelihood of engaging in actual dieting and exercising behaviours. Weight comparisons to friends amplified these effects.” [66]

T131(+). “Whether the target was a friend moderated these effects. When engaging in an upward comparison to a friend, participants had more thoughts of exercising compared to when the target of the upward comparison was not a friend (Y=1.03, P=0.031). When engaging in a downward comparison to a friend, participants also reported more thoughts of dieting (Y=2.68, P=0.006) and exercising (Y=2.13, P=0.024) as compared to when targets were nonfriends.” [66]

T132(-). “During treatment, participants lost an average of 4.4% of initial body weight, and social influence factors were adversely associated with weight loss outcomes. Having more casual friends who were overweight at baseline and being part of a social network with stronger social norms for unhealthy eating predicted poorer weight losses (p’s<.023).” [27]

T133(+). “A person’s chances of becoming obese increased by 57% (95% confidence interval [CI], 6 to 123) if he or she had a friend who became obese in a given interval. Among pairs of adult siblings, if one sibling became obese, the chance that the other would become obese increased by 40% (95% CI, 21 to 60). If one spouse became obese, the likelihood that the other spouse would become obese increased by 37% (95% CI, 7 to 73).” [68]

T134(+). “The sex of the ego and alter also appeared to be important. When the sample was restricted to same-sex friendships (87% of the total), the probability of obesity in an ego increased by 71% (95% CI, 13 to 145) if the alter became obese.” [68]

*3.7 Housekeeping*

Q125(-). “Our weights increase because we have housemaids and we depend on them a lot.” [52]

*3.8 Co-workers*

T135(+): “In a multivariable regression model, greater weight loss was associated with help from a child with eating goals (p=.0002) and co-worker help with physical activity (p=.01).” [64]

*3.9 Pets*

T136(+). “In addition to reinforcing family bonds, these participants felt that exercising with their spouse or close family member provided the accountability needed to maintain good habits. For several participants, pets provided much needed companionship and reason to be active. Pets appeared to be especially important motivators of physical activity for elderly individuals living alone.” [55]

1. **Properties**

*4.1 Tie strength (frequency of contact and feeling of closeness)*

T137. “In order to allow for variable schedules, teams were not necessarily expected to walk as a group, but they were expected to have regular contact in order to serve as motivational resources and walking partners for group members as their schedules permitted.” [50]

T138. “Moreover, having more friends is associated with an improvement in health, while being healthy and prosocial is associated with closer relationships. Specifically, a unit increase in health is associated with an expected 0.45 percentage-point increase in average closeness, while adding a prosocial activity is associated with a 0.46 percentage-point increase in the closeness of one’s relationships.” [60]

T139. “Lower frequencies of family contact were associated with lower fruit variety scores and rare/no contact was similarly negative for both genders. By contrast, decreasing family contact seemed to have limited association with vegetable variety in men whereas weekly contact had a 0.56 unit difference (p ¼ 0.001) in score in women compared with daily family contact.” [61]

T140. “Similarly, unit differences in vegetable variety scores were significantly lower (p ¼ 0.026) for widowed over-50s with infrequent friend contact than for those with frequent contact (-2.02 versus -0.87; both p < 0.001) compared to partnered counterparts-a difference of 1.15 items/day.” [61]

T141. “Results for family contact and vegetable variety were most surprising: weekly contact (versus daily) was significantly positively associated in women, but men showed limited associations.” [61]

T142. “Women who dined more often with healthy eaters reported on average a higher diet quality and a lower body mass index (BMI).” [62]

T143. “The degree to which this behaviour is shared is modulated by the strength of the relationship between the two individuals, with a greater probability of engaging in these behaviours observed when the relationship with the nominated peer is strong relative to when the relationship is weak.” [63]

T144. “Participants in contact with a child helpful with eating goals ≤ once a week (but at least once a month) had borderline greater weight loss compared to those with more frequent contact [-13.9 (±10.7), n=8 vs. -5.6 (±11.2) lbs., n=93, P =.05].” [64]

T145. “Our data suggest that emotionally close social ties (partners, best friends, children, relatives) have a greater association with obesity status at baseline than social contacts with presumably less emotional valence (e.g., casual friends, colleagues).” [27]

*4.2 Degree (number of contacts)*

T146. “Moreover, having more friends is associated with an improvement in health, while being healthy and prosocial is associated with closer relationships. Specifically, a unit increase in health is associated with an expected 0.45 percentage-point increase in average closeness, while adding a prosocial activity is associated with a 0.46 percentage-point increase in the closeness of one’s relationships.” [88]

T147. “Furthermore, increased degree is associated with reduced transitivity, while closeness and transitivity are positively correlated. Thus, as an individual accumulates more alters, the average closeness of their own relationships and of the relationships between the alters in their egocentric network decline.” [88]

*4.3 Size of network*

T148. “Weight gain was associated with having network members with obesity living in the home (p=.048) and increased network size (p=.002).” [64]

T149. “participants reporting social contact with 6 or 7 friends on a weekly basis had a 24% lower mortality risk than those in contact with ≤ 1 friend (HR 0.76, 95% CI 0.58–0.98).” [65]

T150. “During treatment, participants lost an average of 4.4% of initial body weight, and social influence factors were adversely associated with weight loss outcomes. Having more casual friends who were overweight at baseline and being part of a social network with stronger social norms for unhealthy eating predicted poorer weight losses (p’s<.023).” [27]

*4.4 Degree of separation*

T151. “Whereas increasing social distance appeared to decrease the effect of an alter on an ego, increasing geographic distance did not. The obesity of the most geographically distant alters correlated as strongly with an ego’s obesity as did the obesity of the geographically closest alters. These results suggest that social distance plays a stronger role than geographic distance in the spread of behaviours or norms associated with obesity.” [68]

*4.5 Social distance*

T152. “Whereas increasing social distance appeared to decrease the effect of an alter on an ego, increasing geographic distance did not. The obesity of the most geographically distant alters correlated as strongly with an ego’s obesity as did the obesity of the geographically closest alters. These results suggest that social distance plays a stronger role than geographic distance in the spread of behaviours or norms associated with obesity.” [68]
