# Supplementary material for: Social network influences and the adoption of obesity-related behaviours in adults: a critical interpretative synthesis review
Source: BMC Public Health. 2019 Aug 28;19:1178. doi: 10.1186/s12889-019-7467-9 (PMC6712627; doi:10.1186/s12889-019-7467-9)
Supplement: Supplementary file 2 — Coalitions. (DOCX 33 kb) [file 12889_2019_7467_MOESM2_ESM.docx]

Additional file 2. Coalitions

1. **Meso-level**

1.1 Social support

*1.1.1 Peer support*

Q1(+). “My doctors said before that I need to, to walk more … I mean it’s the kind of thing that you sort of – you kind of know anyway really within yourself that you know you’re not doing enough of any kind of exercise. This [study invitation] is what gave me the stimulus if you like to actually get on and do something about it … and even the doctor didn’t even manage to persuade me of that.” [58]

*Coalition: Healthcare professionals (type of tie, meso-level) + peer support (process, meso-level).*

Q2(+). “Several participants suggested external health promoters could provide additional encouragement: ‘Somebody coming in from outside, say doing half an hour at lunchtime just doing a presentation about it or, you know, longer and getting people there and talking about that and saying ‘and we have our in-house person who you know if you want to talk to him, d’you wanna get encouragement from him/her’ that would be great but I think somebody coming in from outside actually would be a good idea.” [58]

*Coalition: Healthcare professionals (type of tie, meso-level) + peer support (process, meso-level).*

*1.1.2 Group support*

T1(+). “During follow-up visits all but one woman in the study agreed that men and women were not active together. In contrast, women reported discussing exercise with other women and joining all-female exercise groups.” [47]

*Coalition: Sport contacts (type of tie, meso-level) + group support (process, meso-level).*

Q3(+). “When I had to reduce weight, I went to a weight loss group at ‘X’… When they realized that I was a cook I was engaged to take part in cooking courses and things like that for overweight people with diabetes who have challenges with their intake…” [49]

*Coalition: Community organizations (type of tie, meso-level) + group support (process, meso-level) + community resources (contextual factor/environmental factor, meso-level).*

T2(-). “Participation and attendance at the pub involve negotiations and a counterbalance of the intake of beer and the health promoting effects of positive social relationships taking place at the pub.” [51]

*Coalition: Community others (type of tie, meso-level) + peer support (process, meso-level) + social norms (contextual factor/socio-cultural factor, meso-level).*

Q4(+). “That’s why I think the group would be kind of cool to get together with… to get together as a group and just share some ideas ...” [55]

*Coalition: Community other (type of tie, meso-level) + group support (process, meso-level).*

*1.2 Homophily*

T3(+). “During follow-up visits all but one woman in the study agreed that men and women were not active together. In contrast, women reported discussing exercise with other women and joining all-female exercise groups.” [47]

*Coalition: Sport contacts (type of tie, meso-level) + homophily (process, meso-level).*

Q5(+). “When I had to reduce weight, I went to a weight loss group at ‘X’… When they realized that I was a cook I was engaged to take part in cooking courses and things like that for overweight people with diabetes who have challenges with their intake…” [49]

*Coalition: Community organizations (type of tie, meso-level) + homophily (process, meso-level) + community resources (contextual factor/environmental factor, meso-level).*

T4(+). “Most of the women agreed that they prefer walking with other women; they feel safer and more comfortable.” [52]

*Coalition: Community (others)(type of tie, meso-level) + homophily (process, meso-level) + social norms (contextual factor/socio-cultural factors, meso-level).*

T5(+). “Persons of the same sex had relatively greater influence on each other than those of the opposite sex.” [68]

*Coalition: Others (type of tie, meso-level) + homophily (process, meso-level) + social norms (contextual factor/socio-cultural factors, meso-level).*

*1.3 Social pressure*

Q6(+). “I probably would pass somebody from my gym somewhere on the streets [… It’s] motivational in the sense that if you don’t go […to the gym] and pass a girl that I haven’t seen in a while ‘hey why I don’t see you in the gym? What’s going on with you?’ and I guess guilt people into coming back. So yeah it’s motivation.” [47]

*Coalition: Sport contacts (type of tie, meso-level) + social pressure (process, meso-level) + motivation (contextual and individual factor/psychosocial factor, micro-level).*

Q7(+). “Cause for instance I sit home and I wouldn’t walk, but if somebody called me and I know I’m in a group and we walkin’ today, I’ll try to walk…cause you don’t wanna let anybody down.” [50]

*Coalition: Community (others) (type of tie, meso-level) + social pressure (process, meso-level).*

Q8(+). “What happened to you the other day? Why didn’t you come walking? And it’s kind of – its sort of an accountability where if you’ve got that moral support from saying ‘What’s up? You missed two days.” [50]

*Coalition: Community (others) (type of tie, meso-level) + social pressure (process, meso-level).*

T6(-). “Participation and attendance at the pub involve negotiations and a counterbalance of the intake of beer and the health promoting effects of positive social relationships taking place at the pub.” [51]

*Coalition: Community others (type of tie, meso-level) + social pressure (process, meso-level) + social norms (contextual factor/socio-cultural factor, meso-level).*

Q9(-). “However, if I go back to the village where my husband comes from, they are country people and they love to bake, cook and it’s lovely. It’s gorgeous and because they know you’re coming for afternoon tea, they’ll have made you the apple pie and they’ll have made you the cakes and if you went in there and tried to start explaining that you don’t eat any of that… In that sort of culture, it just would not be understood, and also you’re interfering with the social norms and you don’t want to do that.” [51]

*Coalition: Community others (type of tie, meso-level) + social pressure (process, meso-level) + social norms (contextual factor/socio-cultural factor, meso-level).*

T7(-). “Participants described food-centric social events as a primary constraint to eating well. Limited entertainment options in these rural communities meant that most activities involved getting together for a snack or meal. Food provision was regarded as a sign of “hospitality” and people felt obligated to eat whatever was offered in social settings (e.g. church, senior centers).” [55]

*Coalition: Community organizations (type of tie, meso-level) + social pressure (process, meso-level) + social norms (contextual factor/socio-cultural factor, meso-level).*

Q10(+). “An element of competition may help… if the progress was recorded and shared between all the people on the scheme, it could possibly have a positive effect…” [58]

*Coalition: Community (others) (type of tie, meso-level) + social pressure (process, meso-level) + motivation (contextual and individual factor/psychosocial factor, micro-level).*

*1.4 Natural communication*

T8(+). “WhatsApp groups comprised of women in the same exercise class could make this social pressure and social support even stronger.” [47]

*Coalition: Sport contacts (type of tie, meso-level) + natural communication (process, meso-level) + personal attitude (contextual and individual factor/psychosocial factor, micro-level).*

*1.5 Social modelling*

Q11(+). “Participants described a program leader who would help in these areas. One expressed a desire for such a person “to motivate us or have the knowledge, but both would be good.” Another described this role as “It’s somebody that will enhance the group to walk.” [50]

*Coalition: Community (others) (type of tie, meso-level) + social modelling (process, meso-level).*

Q12(+). “We have people in my neighbourhood that you can be leaving out at five in the morning, and they’re walking. You can come in at six in the afternoon and there’s another group walking…We have a monthly HOA [homeowners association] meeting— and sometimes in those meetings people just go, “Hey, I saw you walking. Can I join your group?” [53]

*Coalition: Neighbours (type of tie, meso-level) + social modelling (process, meso-level).*

*1.6 Diffusion*

T9(+). “All participants received a Walk Member Handbook with community trail maps and other information sheets about issues of interest.” [50]

*Coalition: Community (others) (type of tie, meso-level) + diffusion (process, meso-level) + knowledge (contextual and individual factor/psychosocial factor, micro-level).*

Q13(+). “That’s why I think the group would be kind of cool to get together with… to get together as a group and just share some ideas…” [55]

*Coalition: Community other (type of tie, meso-level) + group support (process, meso-level).*

T10(+). “Much of this health information was viewed with a degree of scepticism, particularly claims made about food. In contrast to the scepticism levelled at scientific, medical and government information sources, health messages that derived from within participants’ own social network were given more credence.” [55]

*Coalition: Community (others) (type of tie, meso-level) + diffusion (process, meso-level) + knowledge (contextual and individual factor/psychosocial factor, micro-level).*

Q14(+). “Overall the promoters found their booklet ‘was well set out’ and helped them approach participants: ‘It was informative and useful and helped me set out what I needed to do, promote walking to work to the colleagues, and how to approach them and stuff, I thought it was quite good.” [58]

*Coalition: Community (others) (type of tie, meso-level) + diffusion (process, meso-level) + knowledge (contextual and individual factor/psychosocial factor, micro-level).*

1. **Micro-level**

*2.1 Social modelling*

T11(+). “When asked about recommended strategies for encouraging a friend to walk, participants emphasized social support in the form of direct encouragement (e.g., “you can make it”), serving as a role model, and offering to walk with the friend.” [50]

*Coalition: Friends (type of tie, micro-level) + social modelling (process, micro-level).*

T12(+). “In contrast, having tight social networks was viewed as beneficial if friends were “health-conscious” and acted as positive role models.” [55]

*Coalition: Friends (type of tie, micro-level) + social modelling (process, micro-level).*

*2.2 Social comparison*

T13(+). “While describing their motivations for pursuing physical activity, some women explained that witnessing friends and family with long term chronic conditions motivated them to take control of their own health as much as possible.” [47]

*Coalition: Friends or family (type of tie, micro-level) + social comparison (process, micro-level) + critical moments (contextual and individual factors/psychosocial factors, micro-level).*

Q15(-). “I think it’s also about what I know I’m missing out on if I do exercise more. I mean, my friends and family are often going out of an evening, seeing films or going out to dinner or for drinks. I’d have to miss out on all of that if I was prioritising exercise a couple of nights a week, or even just eating really healthily would inhibit my ability to just do things like that. I don’t want to miss out.” [54]

*Coalition: Family (type of tie, micro-level) + social comparison (process, micro-level).*

T14(+). “Watching a family member’s health deteriorate came as a wake-up call to change their own behaviour.” [55]

*Coalition: Family (type of tie, micro-level) + social comparison (process, micro-level) + critical moments (contextual and individual factors/psychosocial factors, micro-level).*

Q16(+). “My grandmother… when I was 13… I was the sole witness to her coronary occlusion which killed her on the spot and I never quite dealt with that so it has left me with a bit of a fear of heart disease and heart problems and seeing how violently they can end your life.” [56]

*Coalition: Family (type of tie, micro-level) + social comparison (process, micro-level) + critical moments (contextual and individual factors/psychosocial factors, micro-level).*

T15(+/-). “In the case of body size, a descriptive norms effect can work through direct comparison so that a person compares himself to others in his social reference group and makes decisions regarding his own status according to that metric.” [45]

*Coalition: Friends or family (type of tie, micro-level) + social comparison (process, micro-level).*

T16(+). “Relative to social comparisons to targets of the same weight, weight-focused comparisons to both thinner and heavier individuals led to increased thoughts of dieting and exercising. Moreover, comparisons to thinner targets also increased the likelihood of engaging in actual dieting and exercising behaviours. Weight comparisons to friends amplified these effects.” [66]

*Coalition: Friends (type of tie, micro-level) + social comparison (process, micro-level).*

*2.3 Social pressure*

T17(-). “Social events involving food were areas where maintaining normal social ties were often more important than attempting to force attention on dietary needs.” [49]

*Coalition: Friends (type of tie, micro-level) + social pressure (process, micro-level) + social events (contextual and individual factors/socio-cultural factors, micro-level).*

Q17(+). “My husband insists that I shouldn’t eat large quantities or any starchy food. My mum always scolds me, but this doesn’t help; she just gets on my nerves. As soon as she sees me eating even the smallest amount of sweets, she’ll start complaining. I can’t say my daughters are indifferent. They’ll remark when I overeat something. Everyone is focused on my diet.” [51]

*Coalition: Family (type of tie, micro-level) + social pressure (process, micro-level).*

T18(-). “This man emphasizes the need to occasionally not adhere to the diet, especially at parties and when with friends.” [51]

*Coalition: Friends (type of tie, micro-level) + social pressure (process, micro-level) + social events (contextual and individual factors/socio-cultural factors, micro-level).*

Q18(-). “Often, when [you are] offered a piece of chocolate, you don’t say that you have diabetes, you take it and eat it just because you don’t want to offend the person and you feel uncomfortable admitting you have diabetes and you shouldn’t eat sweets.” [51]

*Coalition: Family or friends (type of tie, micro-level) + social pressure (process, micro-level).*

Q19(+). “If you have someone that’s going to encourage you, say, “Come on, let’s go, let’s go do this,” or “Come on, it’s only going to take 10 minutes,” or something like that, then you go,“Okay, I’ll do it.” [53]

*Coalition: Family or friends (type of tie, micro-level) + social pressure (process, micro-level).*

T19(+). “One motivator for weight-loss which was raised specifically by young adults was to feel more confident when approaching people they find attractive, and forming intimate relationships.” [54]

*Coalition: Friends (type of tie, micro-level) + social pressure (process, micro-level) + motivation (contextual and individual factors/psychosocial factor, micro-level)*

Q20(-). “When I go to parties or I meet up with friends, I don’t go with my diet, I just eat whatever.” [54]

*Coalition: Friends (type of tie, micro-level) + social pressure (process, micro-level) + social events (contextual and individual factors/socio-cultural factors, micro-level).*

T20(+/-). “Depending on these factors, regularly shared meals, such as family meals, might more or less conform to dietary guidelines although they are often generally perceived as healthy meals.” [62]

*Coalition: Family (type of tie, micro-level) + social pressure (process, micro-level) + social norms (contextual and individual factors/socio-cultural factors, micro-level).*

2.4 Social support

*2.4.1 Peer support*

T21(+). “Asked about who they last heard talking about physical activity, some women explained that they talked with female friends about being active regularly. For these women, peer encouragement was reported to be a strong motivator.” [47]

*Coalition: Friends (type of tie, micro-level) + peer support (process, micro-level) + motivation (contextual and individual factors/psychosocial factors, micro-level) + social norms (contextual and individual factors/socio-cultural factors, micro-level).*

T22(+). “Collective efficacy was most frequently obtained through a respondent’s partner changing their lifestyle, especially in cooking and eating, to make life easier for the partner with diabetes.” [49]

*Coalition: Family (type of tie, micro-level) + peer support (process, micro-level).*

Q21(+). “In the last 2 years things have worsened, because my wife got ill; she was the one who took care of me – she cooked and pleased me with delicious meals, but now she can’t anymore and I have to take care of her and of me (alone). Now, my son started helping me – he buys drugs and provides me with insulin.” [49]

*Coalition: Family (type of tie, micro-level) + peer support (process, micro-level) + critical moments (contextual and eventual factor/psychosocial factor, micro-level).*

Q22(+). “Another participant highlighted the instrumental role played by family members, such as a granddaughter who might say, “Come on Grandma, let’s walk. You know you’re supposed to walk. Now come on, let’s go.” [50]

*Coalition: Family (type of tie, micro-level) + peer support (process, micro-level).*

Q23(+): “My wife makes cakes, but not lately as she doesn’t want to tempt me. But I know they are in the fridge… If we have a barbecue, my family serves me a Diet Coke.” [51]

*Coalition: Family (type of tie, micro-level) + peer support (process, micro-level).*

T23(+). “Meals often involve family gatherings, and negotiations take place related to norms and culture of gender and family life. It appears in the interviews that female partners often play a supportive and active role monitoring their male partner’s diabetes diet underlining gender differences in diabetes.” [51]

*Coalition: Family (type of tie, micro-level) + peer support (process, micro-level).*

T24(+). “However, many men attributed their healthier eating habits to their wives’ food preparation and procurement efforts (e.g. home canning and gardening). Some women also discussed successful compromises during family meal times including serving smaller portions, making healthy recipe modifications, and preparing separate meals.” [55]

*Coalition: Family (type of tie, micro-level) + peer support (process, micro-level).*

T25(+). “Several women cited peer support as a primary motivator for maintaining healthy eating habits.” [55]

*Coalition: Friends or family (type of tie, micro-level) + peer support (process, micro-level) + motivation (contextual and individual factors/psychosocial factors, micro-level).*

Q24(+). “It’s healthy [referring to diet] because I was brought up by my mother who was a very good cook.” [75]

*Coalition: Family (type of tie, micro-level) + peer support (process, micro-level).*

Q25(+). “I used to eat a lot of vegetables when I was at home, cause my wife was an extremely good cook, so we ate really well, I don’t mean gluttony I mean just healthy food.” [75]

*Coalition: Family (type of tie, micro-level) + peer support (process, micro-level).*

T26(+). “Receiving spousal/partner support also reduced mortality risk 19% (HR 0.81, 95% CI 0.66-.99).” [65]

*Coalition: Family (type of tie, micro-level) + peer support (process, micro-level).*

T27(+). “Having a spouse was associated with a better physical health status (B = 1.01), especially for patients with a high income.” [18]

*Coalition: Family (type of tie, micro-level) + peer support (process, micro-level) + socio-economic status (contextual and individual factors/sociodemographic factor, micro-level).*

T28(+). “In a multivariable regression model, greater weight loss was associated with help from a child with eating goals (p=.0002) and co-worker help with physical activity (p=.01).” [64]

*Coalition: Family and co-workers (type of tie, micro-level) + peer support (process, micro-level).*

Q26(+). “My son would say let's walk instead of taking the train or bus.” [64]

*Coalition: Family (type of tie, micro-level) + peer support (process, micro-level).*

*2.4.2 Group support*

Q27(+). “He has performed some navigation work in terms of making judgements of who he might contact in case of emergency, but does not need to do much negotiation as the support that his family provides him with in terms of diet and help in contexts where he does not feel as autonomous, supplements these needs.” [49]

*Coalition: Family (type of tie, micro-level) + group support (process, micro-level).*

Q28(+). “We are trying to exercise together, all of us… We aim to create a large group and include family and kids and socialize very often, so it becomes a big group and better habits.” [49]

*Coalition: Family (type of tie, micro-level) + group support (process, micro-level).*

Q29(+). “What works for me is walking. That’s the only thing that really works. It makes my daughter, who also is headed toward weight problems-it gives her something completely different. She’s trying to do half marathons now. So, we’re both-we see ourselves working into way more activity, even at this age. Even my husband’s getting into it… So, it’s really become a family affair kind of thing… Because that’s the only way I think we’re going to keep it going.” [53]

*Coalition: Family (type of tie, micro-level) + group support (process, micro-level).*

T29(+/-). “Family was a social influence for many, but the effect seemed highly varied. While some found criticisms from their family about being overweight helpful and motivating, others found it discouraging and hurtful.” [54]

*Coalition: Family (type of tie, micro-level) + group support (process, micro-level).*

T30(+). “In addition to reinforcing family bonds, these participants felt that exercising with their spouse or close family member provided the accountability needed to maintain good habits. For several participants, pets provided much needed companionship and reason to be active. Pets appeared to be especially important motivators of physical activity for elderly individuals living alone.” [55]

*Coalition: Family and pets (type of tie, micro-level) + group support (process, micro-level).*

Q30(+). “[If] you wanna eat healthy… you pretty much have to change your friends at that point in time. And like in [this town], who you gonna change your friends to? Ya know, it’s not like you have nine hundred thousand other people that you can go out with and visit with.” [55]

*Coalition: Friends (type of tie, micro-level) + group support (process, micro-level).*

Q31(+). “…my health…[is] my family…My children and husband, and our whanau whanui (tribal family) … our wellbeing is whanau (family)…[when] someone else is not well in our family, that has an impact…on our health…I’m connected to those people and our children…the heavier we are collectively, the better off we are individually…” [57]

*Coalition: Family (type of tie, micro-level) + group support (process, micro-level).*

T31(+). “By contrast, decreasing family contact was associated with lower scores among over-50s with infrequent friend contact such that rare/no contact showed a -1.01 unit difference (p < 0.05) compared to daily contact. Differences in friend contact of one vegetable item/day were significant (p ¼ 0.056) for adults with rare/no family contact.” [61]

*Coalition: Family (type of tie, micro-level) + group support (process, micro-level).*

T32(+). “Participants reporting family support had a 19% lower risk of mortality as compared to participants reporting no family support (P=.01).” [65]

*Coalition: Family (type of tie, micro-level) + group support (process, micro-level).*

*2.5 Homophily*

T33(+). “Asked about who they last heard talking about physical activity, some women explained that they talked with female friends about being active regularly. For these women, peer encouragement was reported to be a strong motivator.” [47]

*Coalition: Friends (type of tie, micro-level) + homophily (process, micro-level) + motivation (contextual and individual factors/psychosocial factors, micro-level) + social norms (contextual and individual factors/socio-cultural factors, micro-level).*

T34(+). “Most of the women agreed that they prefer walking with other women; they feel safer and more comfortable.” [52]

*Coalition: Friends and family (type of tie, micro-level) + homophily (process, micro-level) + social norms (contextual and individual factors/socio-cultural factors, micro-level).*

T35(+). “More precisely, women and their most important eating companions tended to be similar in diet-related factors such as diet quality and eating styles as well as in BMI.” [62]

*Coalition: Friends and family (type of tie, micro-level) + homophily (process, micro-level).*

T36(-). “These results indicated that an unhealthy social eating environment might be a risk factor for the development of unhealthy eating patterns and obesity.” [62]

*Coalition: Friends and family (type of tie, micro-level) + homophily (process, micro-level).*

T37(+). “The results of this study show that the probability of engaging in regular exercise or eating a healthy diet is higher when individuals have friends who also engage in these behaviours.” [63]

*Coalition: Friends (type of tie, micro-level) + homophily (process, micro-level).*

Q32(+). “What works for me is walking. That’s the only thing that really works. It makes my daughter, who also is headed toward weight problems-it gives her something completely different. She’s trying to do half marathons now. So, we’re both-we see ourselves working into way more activity, even at this age. Even my husband’s getting into it… So, it’s really become a family affair kind of thing… Because that’s the only way I think we’re going to keep it going.” [53]

*Coalition: Family (type of tie, micro-level) + homophily (process, micro-level).*

T38(+). “During treatment, participants lost an average of 4.4% of initial body weight, and social influence factors were adversely associated with weight loss outcomes. Having more casual friends who were overweight at baseline and being part of a social network with stronger social norms for unhealthy eating predicted poorer weight losses (p’s<.023).” [27]

*Coalition: Friends (type of tie, micro-level) + homophily (process, micro-level) + social norms (contextual and individual factors/socio-cultural factors, micro-level).*

T39(+). “Persons of the same sex had relatively greater influence on each other than those of the opposite sex”. [68]

*Coalition: Friends and family (type of tie, micro-level) + homophily (process, micro-level) + gender (contextual and individual factors/sociodemographic factor, micro-level).*

T40(+). “The sex of the ego and alter also appeared to be important. When the sample was restricted to same-sex friendships (87% of the total), the probability of obesity in an ego increased by 71% (95% CI, 13 to 145) if the alter became obese.” [68]

*Coalition: Friends (type of tie, micro-level) + homophily (process, micro-level) + gender (contextual and individual factors/sociodemographic factor, micro-level).*

*2.6 Natural communication*

Q33(+). “…found myself you know doing the walking home without having written it down and you know having told several people – I mean telling people that that’s what you’re doing actually makes you hold to it even more than if you, if I’d written it down.” [58]

*Coalition:* *Friends and family (type of tie, micro-level) + natural communication (process, micro-level).*

*2.7 Isolation*

Q34(+). “However, you get fed up and tired; so, when I have the opportunity to be alone and take it easy, I relax and do things I like to do. I also feel that I have better control of the diabetes. No outside negative influence.” [51]

*Coalition: Individual (type of tie, micro-level) + isolation (process, micro-level).*

T41(-). “Several elderly women also discussed the negative consequences of living alone on their diets. Without family members around, eating decisions were primarily based on convenience and several participants reported having no incentive to make dietary improvements at their advanced age.” [55]

*Coalition: Individual (type of tie, micro-level) + isolation (process, micro-level).*

T42(-). “We found that being single or widowed was associated with a lower variety score, particularly vegetable variety, and associations were enhanced when combined with male gender, living alone or infrequent friend contact.” [61]

*Coalition:* *Individual (type of tie, micro-level) + isolation (process, micro-level) + gender (contextual and individual factors/sociodemographic factor, micro-level).*
